# WholePathwayScope: a comprehensive pathway-based analysis tool for high-throughput data

**DOI:** 10.1186/1471-2105-7-30

**Published:** 2006-01-19

**Authors:** Ming Yi, Jay D Horton, Jonathan C Cohen, Helen H Hobbs, Robert M Stephens

**Affiliations:** 1 Advanced Biomedical Computing Center, National Cancer Institute-Frederick/SAIC-Frederick Inc., Frederick, MD 21702, USA; 2 McDermott Center for Human Growth and Development, University of Texas Southwestern Medical Center at Dallas, TX 75390-9046, USA; 3 Departments of Internal Medicine and Molecular Genetics, University of Texas Southwestern Medical Center at Dallas, TX 75390-9046, USA; 4 The Howard Hughes Medical Institute, University of Texas Southwestern Medical Center at Dallas, TX 75390-9046, USA

## Abstract

**Background:**

Analysis of High Throughput (HTP) Data such as microarray and proteomics data has provided a powerful methodology to study patterns of gene regulation at genome scale. A major unresolved problem in the post-genomic era is to assemble the large amounts of data generated into a meaningful biological context. We have developed a comprehensive software tool, WholePathwayScope (WPS), for deriving biological insights from analysis of HTP data.

**Result:**

WPS extracts gene lists with shared biological themes through color cue templates. WPS statistically evaluates global functional category enrichment of gene lists and pathway-level pattern enrichment of data. WPS incorporates well-known biological pathways from KEGG (Kyoto Encyclopedia of Genes and Genomes) and Biocarta, GO (Gene Ontology) terms as well as user-defined pathways or relevant gene clusters or groups, and explores gene-term relationships within the derived gene-term association networks (GTANs). WPS simultaneously compares multiple datasets within biological contexts either as pathways or as association networks. WPS also integrates Genetic Association Database and Partial MedGene Database for disease-association information. We have used this program to analyze and compare microarray and proteomics datasets derived from a variety of biological systems. Application examples demonstrated the capacity of WPS to significantly facilitate the analysis of HTP data for integrative discovery.

**Conclusion:**

This tool represents a pathway-based platform for discovery integration to maximize analysis power. The tool is freely available at .

## Background

In today's post-genomic era, the sequencing projects and the development of High Throughput (HTP) technologies such as microarray and proteomics provide great opportunities to uncover and explore the complexity of biological problems using systems biology. HTP technologies have provided a powerful approach to address a diverse array of biological questions by allowing analysis of the complete transcriptional and translational repertoire of cells or tissues. Pathologically identical tumors can be differentiated into clinically meaningful subgroups by microarray analysis [1-4], and new pathways perturbed in disease states have been identified using microarray analysis [5-7]. Expression arrays also reveal new participants in biological pathways [8-11], new gene targets for pharmaceutical agents, and new functions of genes [12].

Today, the use of DNA microarrays is increasingly widespread and affordable and great expectations have been placed on technological advances in proteomics. However, analyzing the enormous quantity of data generated from such HTP experiments remains a major challenge. A variety of software tools are available to extract and analyze HTP data that primarily focus on microarray data. Two major strategies used are: 1) unsupervised clustering, in which genes are clustered according to changes in expression pattern with no accommodation for biological context, and 2) supervised classification, in which genes are classified according to an underlying or pre-known biology. Numerous existing microarray analysis tools such as GeneCluster [13], TreeView [14], TM4 [15]; and GeneSpring [16] primarily use clustering algorithms, which require significant user effort to connect with biological information. Current HTP data analysis methods, which are primarily based on the computation of data values for each individual gene, such as clustering and classification (Hierarchical, Principle Component Analysis [17], and Significance Analysis of Microarray [18]), do provide great insights in many aspects of experimental analysis. However, a more comprehensive way to integrate and analyze HTP data in the context of biological pathways and networks has become the current need in both academics and industry. As the amount of HTP data has increased and more insightful analysis approaches have been identified, the exploration of the underlying gene regulatory and biochemical networks of pathways to analyze data derived from a variety of HTP technologies has become one of the major challenges in the fields of bioinformatics and computational biology.

Many software tools capable of analyzing HTP data within the context of biological pathways have been developed [19-22]. Recently released commercial software packages including PathwayAssist™ [23], PathArt [24], Ingenuity Pathways Analysis tool [25], MetaCore [26] also compete in the field of pathway-based HTP analysis. These tools provide an assortment of interfaces for the visualization of gene networks, natural language processing (NLP) extracted, or hand-curated biological pathway/association network databases and accept gene-list based data input. Each of these tools has one or more unique features that distinguish it from others. Some open source or publicly accessible software, such as GenMAPP [27], Cytoscape [28], Pathway Processor [29] and ViMac [30], display microarray data within the context of pathways annotated in the Kyoto Encyclopedia of Genes and Genomes (KEGG) pathways [19,22], and provide statistical assessment of the reliability of each differentially expressed gene [29]. However, one of the limitations of these tools is the inability to handle multiple datasets simultaneously in an intuitive way. There is a need for more flexibile and comprehensive HTP data analysis software tools in the public domain that are accessible to the academic community and can provide a suite of utilities to analyze HTP data in biological contexts, such as pathways.

To facilitate the simultaneous analysis and comparison of multiple HTP experiments in the context of biological pathways and association networks, and allow pattern extraction of a selected gene list with biological themes, we developed a stand-alone, Windows-based software tool called WholePathwayScope, or WPS. This software program not only provides many unique ways to analyze and visualize HTP data, but also combines advantages of clustering methodology with a more intuitive pathway or association network-based analysis, and many other features that allow for more comprehensive data analysis.

## Implementation

### WPS provides a pathway-based platform for integrative data analysis

WholePathwayScope or WPS is a software tool that displays HTP data in user-defined or stored gene groups or pathways. The program incorporates a suite of pre-defined biological pathways and allows for the construction of additional user-defined pathways or collections of genes. It also allows generation of biological association networks composed of gene-pathway/term relationships, which can be further manipulated and converted to subnetworks, gene-gene, or pathway/term-pathway/term networks. Results from multiple HTP experiments can be visualized simultaneously, both as summary data from multiple pathways (WSCP) and as detailed data for individual pathways (PSCP). Results can be displayed numerically and can be color-coded according to user-defined criteria to facilitate visual analysis. The program also offers statistical evaluation of global functional category (GO term, pathway etc.) enrichment in a user's gene list, or of user-defined pattern enrichment of choice genes that have been color-coded with HTP data directly.

The program is written in Microsoft Visual Basic 6 and runs in the Microsoft Windows environment. It utilizes Microsoft Access Databases including the internal databases for gene annotations, pathways, gene ontology and disease association information as well as designated criteria (CRI) files for HTP data. Pathways and association networks are created and presented in windows or graphical user interfaces (GUIs), and stored and accessed either in individual files or dynamically within Microsoft Access Databases. Users control the program through a user interface involving GUIs provided from a series of panels, menus and windows. There is also an extensive context-sensitive help system.

### Internal database for gene, pathway, and disease annotation

WPS includes an internal database for integrating gene annotation information from both mouse and human genomes. Annotation covered includes GenBank accession numbers (GenBank IDs), Unigene IDs, Locuslink IDs (Now Entrez Gene), Gene Symbols, Aliases [31], SwissProt IDs (Protein IDs) [32], and disease information from both Genetic Association Database [33] and a partial MedGene Database [34]. It also carries pathway/term information including KEGG [19,22,35], Biocarta [36], CGAP [37] and Gene Ontology information [38-40] for the purpose of gene-term association network generation and Fisher's exact test.

### Designed Files in WPS

**Figure 1:**
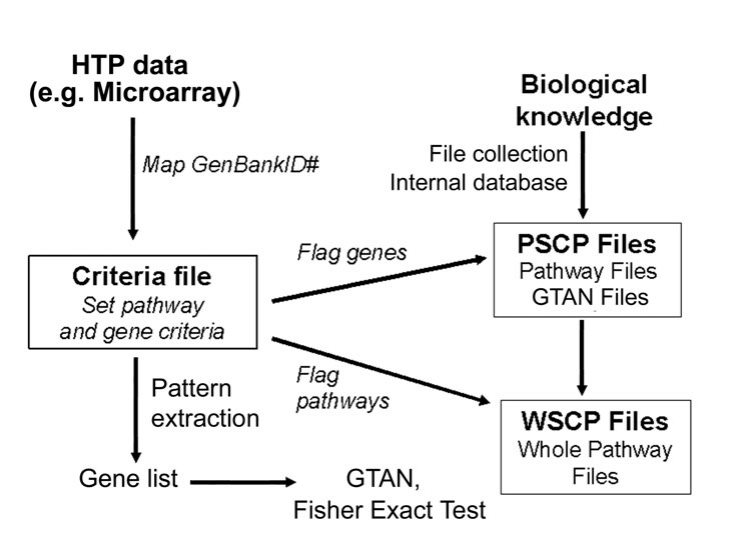
Overview of WholePathwayScope (WPS): schematic work flow for WPS basic data and file processing. WPS imports the data from the microarray file to the criteria (CRI) file, from which gene lists can be obtained by pattern extraction and then subjected to statistical evaluation by Fisher's exact test or to generate a GTAN. The GenBank IDs of genes in microarray datasets and pathway files (PSCP and WSCP files) are mapped to BaseGenBankIDs. The user then sets the criteria by which the gene tags and pathway tags in the PSCP and WSCP files are to be colored. PathwayScope Files (PSCP) including the GTAN (gene-term association network) files and WholeScope Files (WSCP) are either provided or created by the user. WPS integrates the data from the CRI files with the PSCP and WSCP files so that genes and pathways are flagged according to the specifications of the user.

The overall program layout is described in Figure 1. Three types of files with different formats have been developed to display the HTP data. These three file types lay out the pathways being visualized, interconnections amongst those pathways, and data filtering and color criteria for the HTP data respectively. The first file type is the PathwayScope File (PSCP), which is graphical presentation of many metabolic pathways and gene groupings. PSCP files contain the identifiers (gene tags) for each gene in a group of related genes or in a pathway, which is colorable (see PSCP file examples in Figure 4 and 5). PSCP files can be customized and created by users or created dynamically from the internal database. For Biocarta pathways, the pathway graphs can be also visualized in a separate internet browser and data of selected genes in CRI files (see below) can be highlighted in the graphs as well (see Additional file 1 for screenshot).

The second file type is the WholeScope File (WSCP file), which is composed of a series of pathway tags. Each pathway tag in the WSCP file is linked to a PSCP file saved in a user's desktop or represents a term (either a pathway or GO term in the internal database), which is also colorable like gene tags. The PCSP file can be accessed if it is linked by a pathway tag or a new PSCP file(s) can be dynamically created if the pathway tag represents a database associated term from the WSCP file by clicking on the pathway tag. Global changes in expression levels of genes in each pathway or term can be indicated by setting criteria in CRI files (see below) to color code the pathway tags (see WSCP file example in Figure 3).

**Figure 2:**
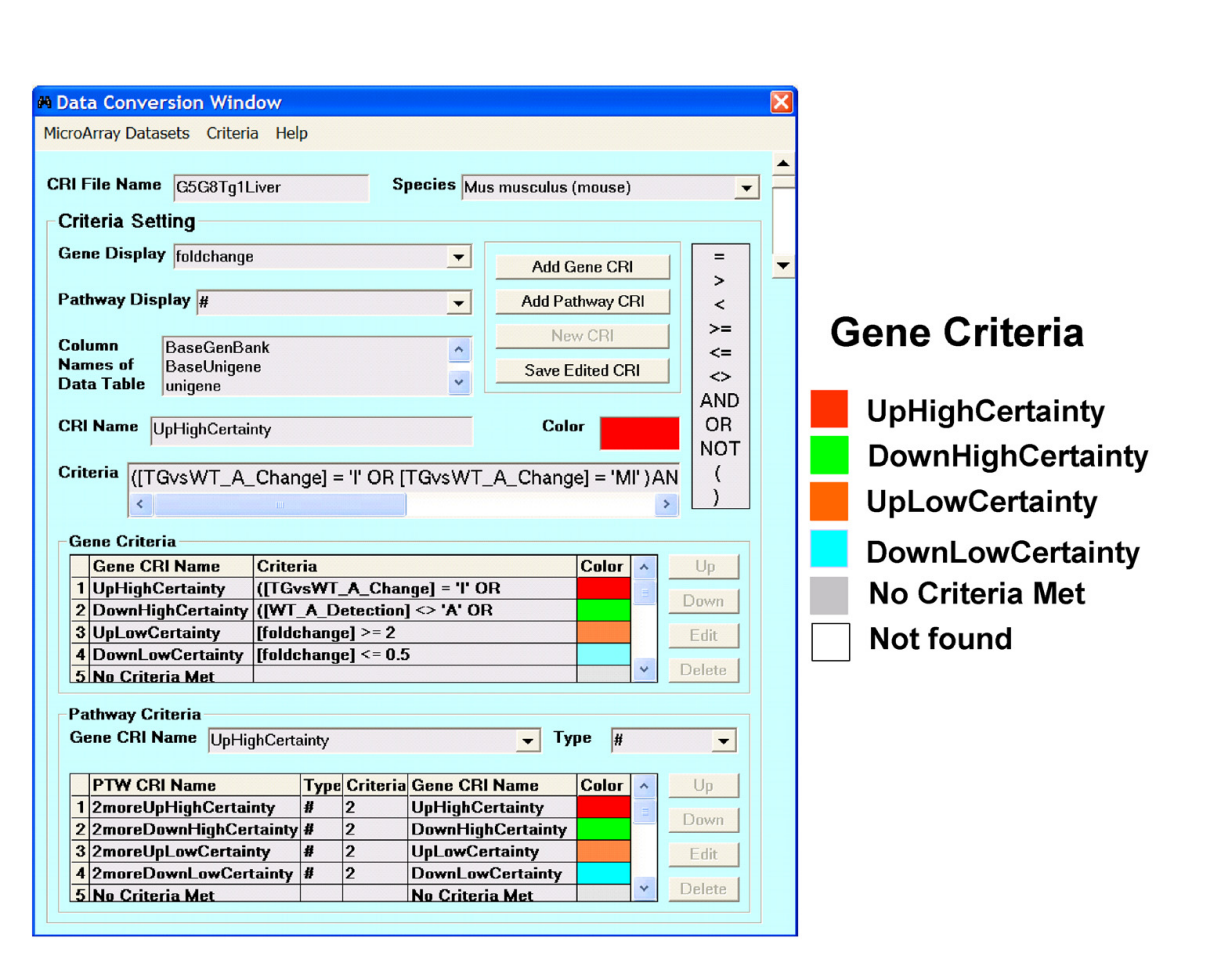
Example of a CRI file created for analysis of microarray data from the *G*5*G*8^*Tg *^mice, shown in the Data Conversion Window. The pathway level criteria (PTW CRI Name) are based on the criteria selected at gene level (Gene CRI Name).

The third file type is the criteria (CRI) file, which is used to enter the user-defined color criteria and HTP data (see Additional files 35–50 for examples of raw data files used in this manuscript). Each CRI file is a Microsoft Access file that contains a HTP (e.g. microarray) dataset, the mapped gene identifiers (BaseGenBankID) for each microarray element, and the user-defined color criteria for the PSCP and WSCP files (see Additional files 19–34 for examples of CRI files used in this manuscript). HTP datasets are converted to CRI files in the program from Excel files (Microsoft, Inc) containing the HTP data through a Data Conversion Window (Fig. 2). WPS can be used for any high-throughput data as long as it is formatted as spreadsheets in Excel files and contains one of the three types of standard gene or protein identifiers including GenBank Accessions, Unigene IDs, or SwissProt IDs (see Additional file 2 or our program demo web page [41] for the file format and procedure for conversion into CRI files). For genes without available standard IDs, they can also be included into CRI files with user-assigned identifiers and used for analysis such as Pattern Extraction (see below). The HTP data in an Excel file can be an individual dataset, or combined multiple datasets in a single file (i.e. in Stanford format [42]). In the latter case, the user can select an appropriate data column to build color criteria specifically for one dataset in the whole file as individual CRI file, or multiple data columns to build color criteria even for multiple datasets within a single CRI file (see the Additional file 3 for more details on the three file types in WPS).

### Data analysis using WPS

Once a series of PSCP, WSCP and CRI files have been loaded into the program, the user has the option of proceeding along several analysis courses. Some of the features available for this analysis are described briefly below. The result section of this manuscript illustrates the program using real data examples to describe some scenarios to apply the program for data analysis. In addition, a set of tutorial movies and illustration image files may be obtained from our program demo web page [41] for many major features and general usage of the program (see additional files 15, 16, 17, 18 for some examples of demo movies).

### Pattern extraction of a list of genes using color cue templates for biological themes

In CRI files, users can define criteria to color code specific categories of behaviors of genes in datasets (e.g. red color for no less than 2 fold change of genes, or green color for genes flagged as down-regulated genes etc.). This kind of criteria definition can be used to extract a gene list of genes matching a specific pattern of such criteria across one or more CRI dataset files.

Pattern extraction can work in two ways: global pattern extraction across datasets, or local or pathway PSCP file scoped pattern extraction. The extracted gene list can be immediately copied and pasted to other utility windows for further analysis (see Fig. 6, 7, 8 and Additional file 4 for illustrative examples).

### Generation and manipulation of Gene-Term Association Network (GTAN) to explore gene-pathway or gene-term relations

Using an input or filtered gene list, such as a list of genes derived from clustering analysis from other programs or pattern extraction in WPS, the associated pathways or GO terms can be identified from the internal database or user-defined PSCP files. These results are listed into the result table in a gene-term pairwise format (Fig. 8). Then, the pairwise relationships between genes and their associated pathways or GO terms in the results table can be used to generate a gene-term association network (GTAN) within a PSCP file and is illustrated in a graphical view (Fig. 9). The generation of such a network is based on the Scalable Vector Graphics (SVG) technology [43], a standard for describing the layout of two-dimensional graphics in XML. The gene-term association network can be manipulated and filtered for the purpose of different analysis needs in many ways (see a concrete example illustrated in Fig. 9, 11, 12, 14 and see Additional file 5 for more detailed description of this feature). In addition, genes associated with disease terms, which were derived from Genetic Association Database and MedGene Database and included in the internal database described above, can be highlighted and selected from the network for further analysis and network manipulation (see Additional file 6 for screenshot).

### Fisher's exact test for biological significance of gene lists and pathway-level pattern enrichment of high-throughput data

The Fisher's exact test is performed based on 2 × 2 contingency tables (whether a gene is in the given list or not vs whether this gene is associated with a pathway/term or not; see Additional file 7 for illustration of an example of 2 × 2 contingency table). Similar to EASE [44], Fisher's exact test p-values are computed for each term in a chosen system, which are then ranked from smaller to larger values, to estimate the statistical significance and enrichment of global functional categories (GO terms, pathways etc.) within a given system for a list of genes of a user's interests or that match a pattern. The biological themes of the gene list can be rapidly retrieved from GO system and Biocarta and KEGG pathway collections as top ranked terms or pathways based on the Fisher's exact test p-values (see Figure 7 and Additional file 8 for a concrete example).

In contrast to the global functional statistical estimation of a gene list, within a PSCP file being analyzed and colored by CRI file(s), the statistical significance and enrichment of genes with certain user-defined criteria, can be also estimated by Fisher's exact test for the corresponding CRI file(s) (see Additional file 9 for a screenshot illustration of this feature).

### Other utilities: information search and dataset manipulation

Within the information search window, one can type in a keyword (e.g. Gene Name, GenBankID etc.) to search for its relevant information, including annotation information, as well as associated disease information from the internal database. Two dataset manipulation utilities are available to conveniently manipulate the size of data files so that one or more subsets of a dataset, or sum of multiple datasets can be used for further analysis: 1. Sorting a dataset into pathway/term scoped "sub-datasets" based on PSCP files, or pathways/terms in the internal database (see Additional file 10 for screenshot); 2. Merge data files.

## Results

Some of the features of WPS are illustrated using experimental data in the following section. Although only microarray data is utilized, any source of HTP data would be equally suited to the analysis.

### Comparison of multiple datasets within multiple pathways

WholePathwayScope displays HTP data within a biological context. Figure 1 provides an overview of the basic work flow of the WPS program for data and file processing. Microarray data or other HTP data are entered into criteria (CRI) files in which the parameters for analysis are set. The data are then loaded into the PSCP/WSCP files either from the provided file collections or from the internal database, and those genes and pathways that meet user-defined criteria are flagged. In addition, gene lists can be extracted from CRI files for further Fisher's exact test analysis or creation of GTAN. Figure 2 provides an example of the data conversion window, in which gene and pathway criteria are entered. For each gene, GenBank accession numbers that correspond to the gene are collated and given a single BaseGenbank ID number.

By way of example, the WPS program was used to compare gene expression profiles between wild-type mice and two strains of genetically-modified mice that either express high levels of *ABCG5 *and *ABCG8 *(*G5G8 *^*Tg *^mice) or no *ABCG5 *or *ABCG8 *(*G5G8 *^-/- ^mice) [45,46] also see Additional file 11 for a description of material and data preparation). *ABCG5 *and *ABCG8 *encode ABC half transporters that heterodimerize to limit the intestinal absorption of dietary sterols and to promote the secretion of sterols from the liver into bile [47].

**Figure 3:**
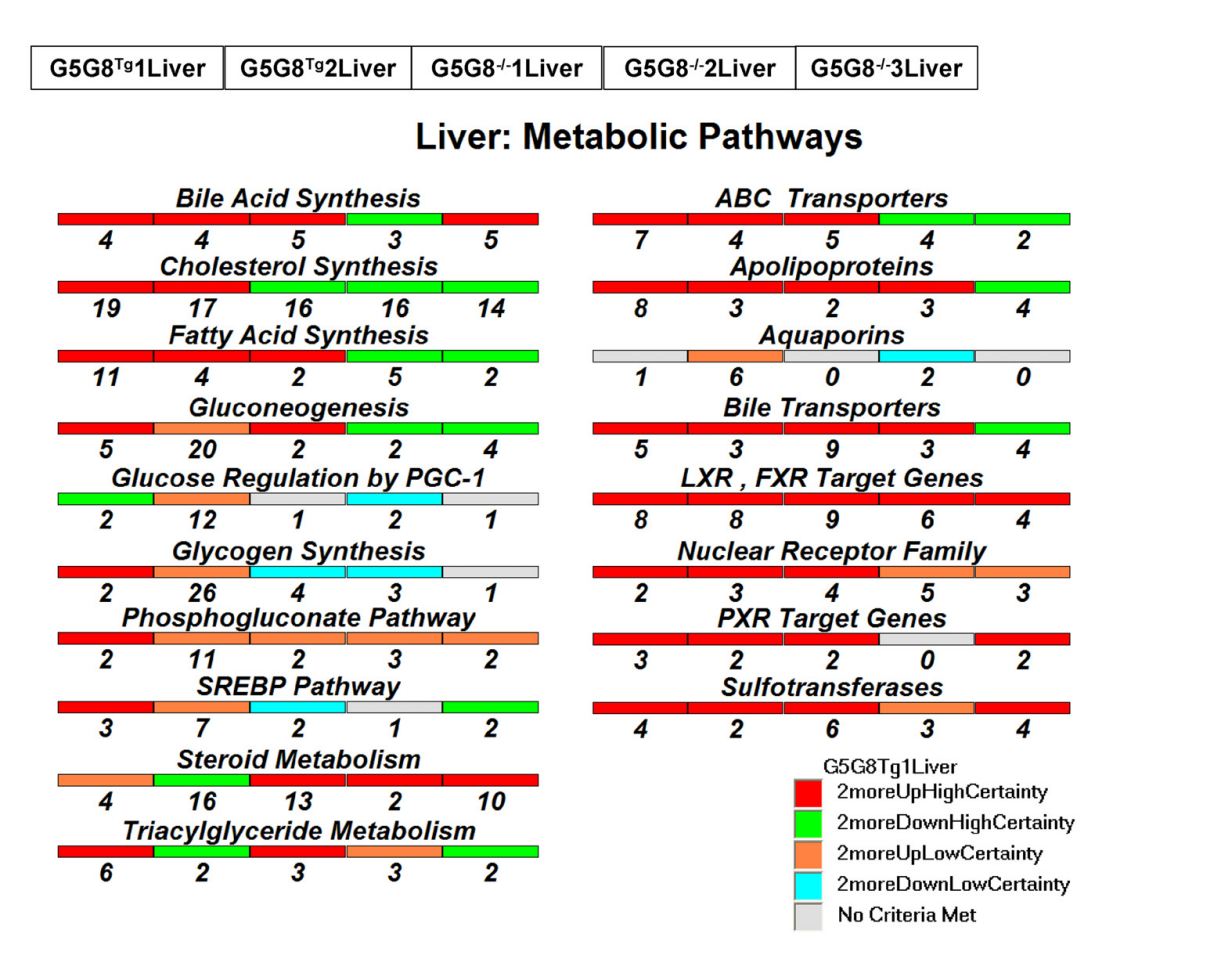
An example of WSCP files in which multiple microarray datasets were analyzed simultaneously. A WSCP file that included a collection of metabolic pathways and gene families with relevance to lipid and glucose metabolism was analyzed using data from the five different microarray datasets (see Additional file 11 for description of material and data preparation). Pooled hepatic mRNA isolated from two sets of *G*5*G*8^*Tg *^mice (G5G8^Tg1^Liver, G5G8^Tg2^Liver) and three sets of *G*5*G*8 knockout mice (G5G8^-/-1^Liver, G5G8^-/-2^Liver, G5G8^-/-3^Liver) were hybridized to Affymetrix chips. Each pathway tag is divided into boxes that are tandemly arrayed in the same order as shown at the top of the figure. Once the data is loaded from each of the experiments, the boxes are colored according to the criteria set by the user. The numbers displayed next to the pathway tags (or divided boxes) are numbers of genes matched with pathway criteria-based gene criteria. (Note: if a single CRI is loaded, the whole pathway tag will be colored by this CRI).

To compare gene expression patterns in the *G5G8 *^*Tg *^and *G5G8 *^-/- ^mice and assess the reproducibility of the microarray results, microarray datasets from five expression array experiments (two from *G5G8 *^*Tg *^mice and three from *G5G8 *^-/- ^mice) were analyzed simultaneously in a WSCP file, which includes a subset of the biochemical pathways and gene families involved in lipid metabolism (Fig. 3). If two or more genes in a pathway were significantly up-regulated or down-regulated (designated "UpHighCertainty" or "DownHighCertainty" in Table 1, respectively) for a dataset, the corresponding divided box of the pathway tag was colored red or green, respectively. Comparison of the data from the two experiments from the *G5G8 *^*Tg *^mice demonstrated consistent results for some pathways (e.g. cholesterol synthesis and bile acid synthesis) but not for other pathways (e.g. glycogen synthesis).

**Figure 4:**
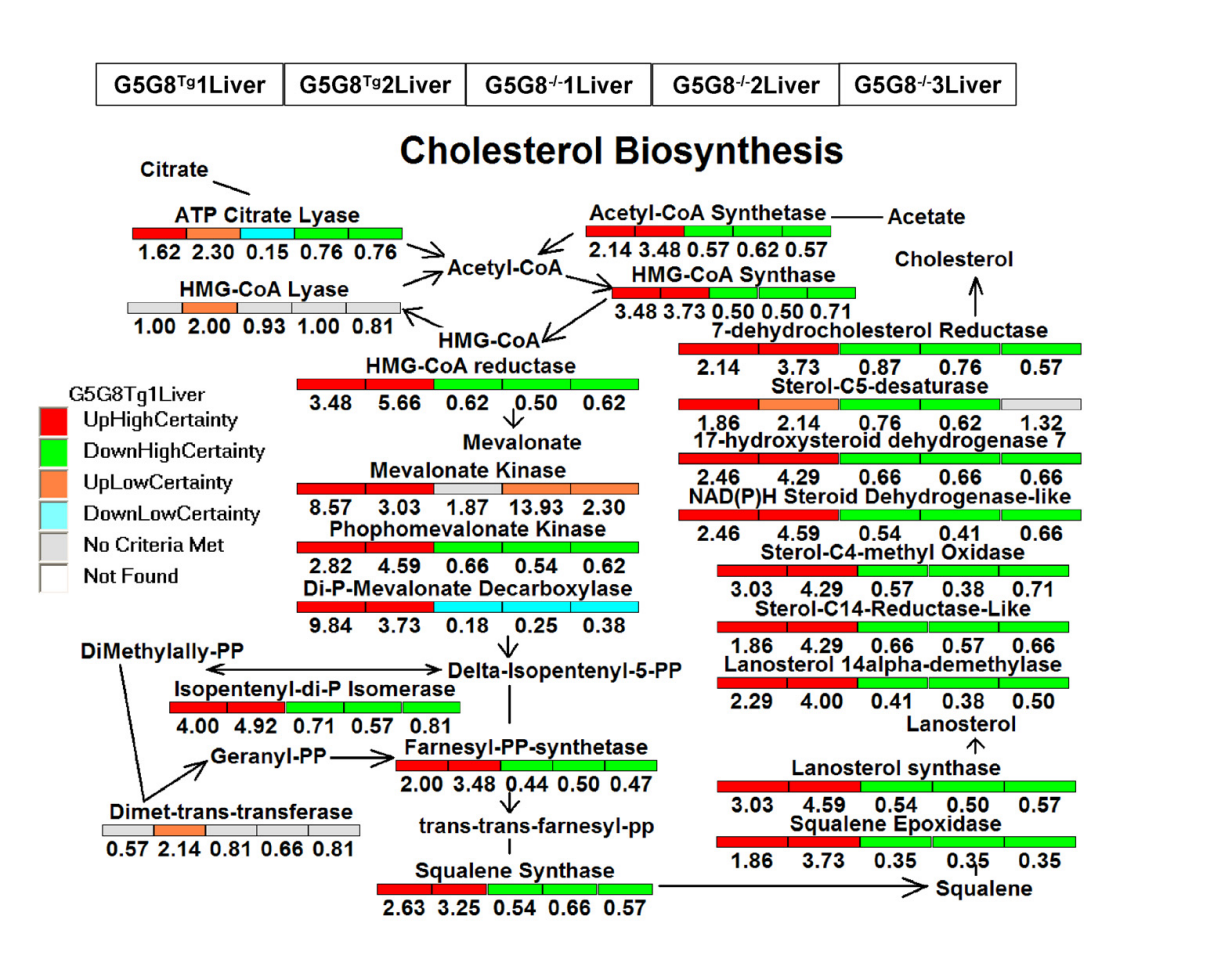
An example of PSCP files in which multiple microarray datasets were analyzed simultaneously. When the pathway tag labeled as 'Cholesterol Synthesis' in Fig. 3 is clicked, the PSCP file is accessed. The numbers displayed proximal to the gene tags (or divided boxes) are the fold changes of the specific genes.

When the WSCP file window is displayed in the program, it is interactive. Each pathway tag in the WSCP file links to a PSCP file or a term in the internal database. For example, clicking on one of its divided boxes of the pathway tag "Cholesterol Synthesis" (Fig. 3) will open a new window with the details of the pathway, including the expression levels of the individual genes (Fig. 4). Many genes in the cholesterol biosynthetic pathway were colored red indicative of up-regulation in both datasets from the *G5G8 *^*Tg *^mice (Fig. 4). These genes were expressed at significantly lower levels (colored green) in samples from the *G5G8 *^-/-^, confirming that differences in expression levels of *ABCG5 *and *ABCG8 *have a significant impact on cholesterol biosynthesis in the liver. When cholesterol synthesis was measured in these two genetically-modified strains of mice, it was found to be increased in the transgenic animals and decreased in the knockouts, verifying the biochemical changes seen in these experiments [45,46].

**Figure 5:**
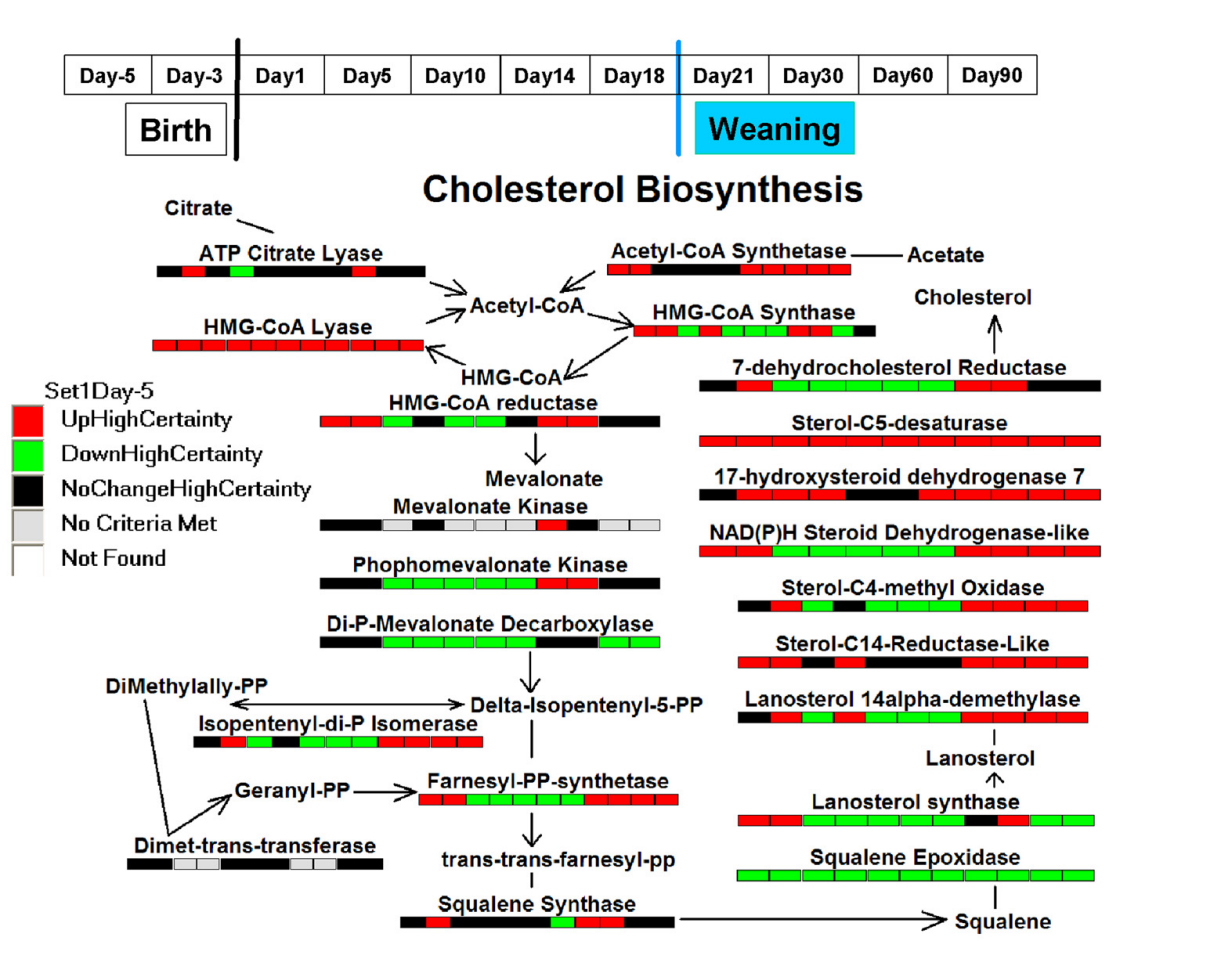
An example of PSCP files in which multiple microarray datasets were analyzed simultaneously. The PSCP file for "Cholesterol Synthesis" pathway analyzed for the data from 11 different microarray datasets or CRI files representing a time course experiment (see Additional file 11 for description of material and data preparation). Pooled hepatic mRNA were isolated from female wild type mice sacrificed at different time points during fetal and post-natal development indicated. The time point at 9 day before birth was used as the reference level of mRNA. "Day-5" and "Day-3" indicates 5 days or 3 day prior to birth, respectively.

To assess the ontogeny of expression of genes in the cholesterol biosynthetic pathway, we analyzed microarray datasets from livers of wild-type mice or embryos sacrificed at different time points during development (Fig. 5). In utero, most cholesterol for the developing mouse is derived from endogenous synthesis, as reflected by the increased expression of many genes in the biosynthetic pathway at day -5 and day -3. Most of these mRNAs decreased within 24 h of birth, which correlates with the initiation of nursing. Many hepatic mRNAs in the group were increased between postnatal days 18 and 21, when the pups transition from consuming a high cholesterol diet (milk) to a low cholesterol chow diet (0.02% cholesterol by weight). During this time period, endogenous cholesterol synthesis compensates for the reduced dietary intake of cholesterol associated with weaning.

### Pattern extraction and statistical evaluation of gene lists for biological themes

**Figure 6:**
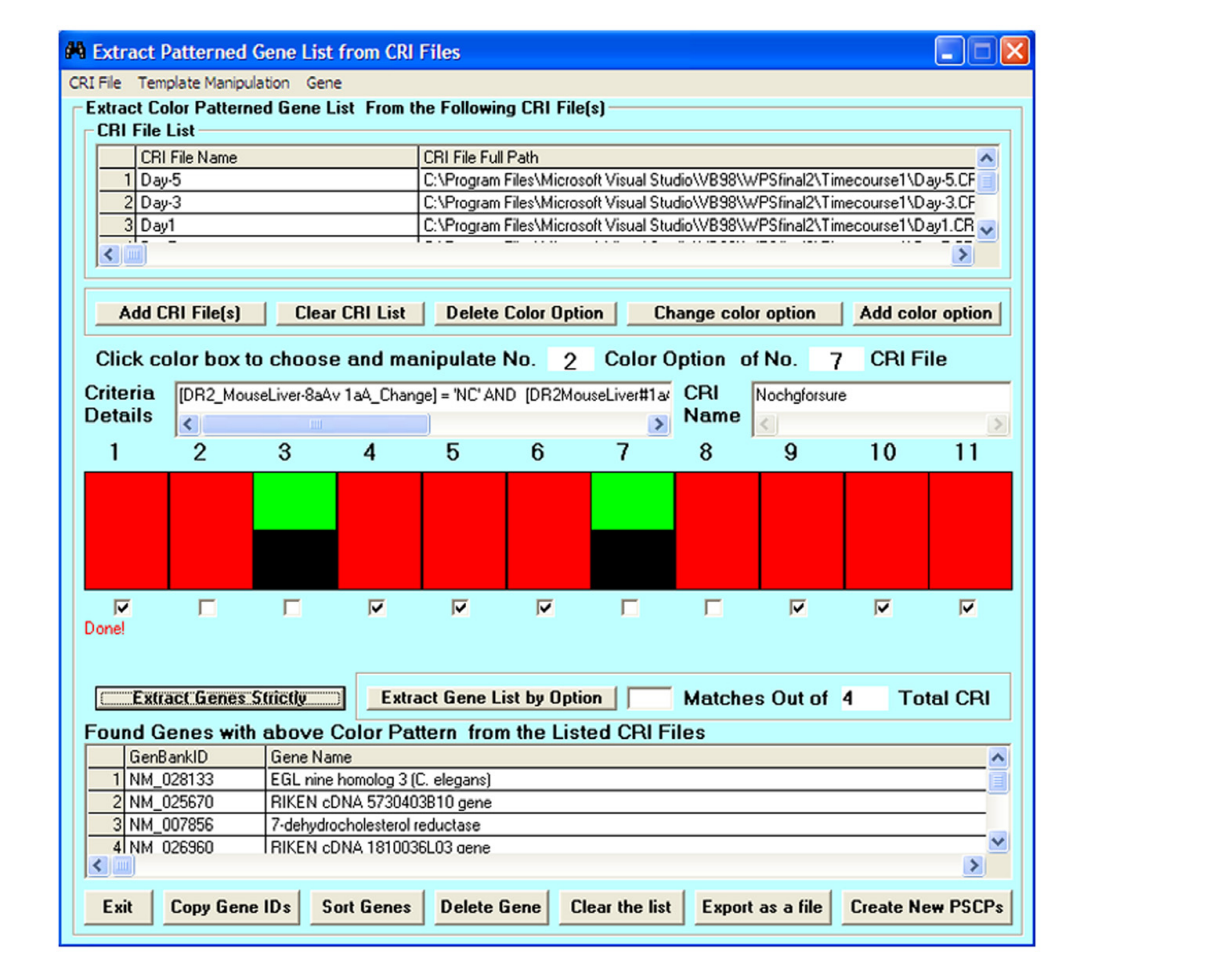
The <Extract Patterned Gene List from CRI Files> window for pattern extraction. CRI files from time course experiments were entered into this window. The template pattern was set as shown in middle of the window. Totally 11 color blocks represented 11 CRI files of time course experiments. The small check boxes under the color blocks are to determine if the corresponding color block or CRI template is ignored If the underneath check box is checked. The multiple color templates were set for No. 3 (Day 1) and No. 7 (Day 18) CRI files as indicated by vertically discrete boxes (green and black boxes). Extracted genes were entered into the table for further processing.

**Figure 7:**
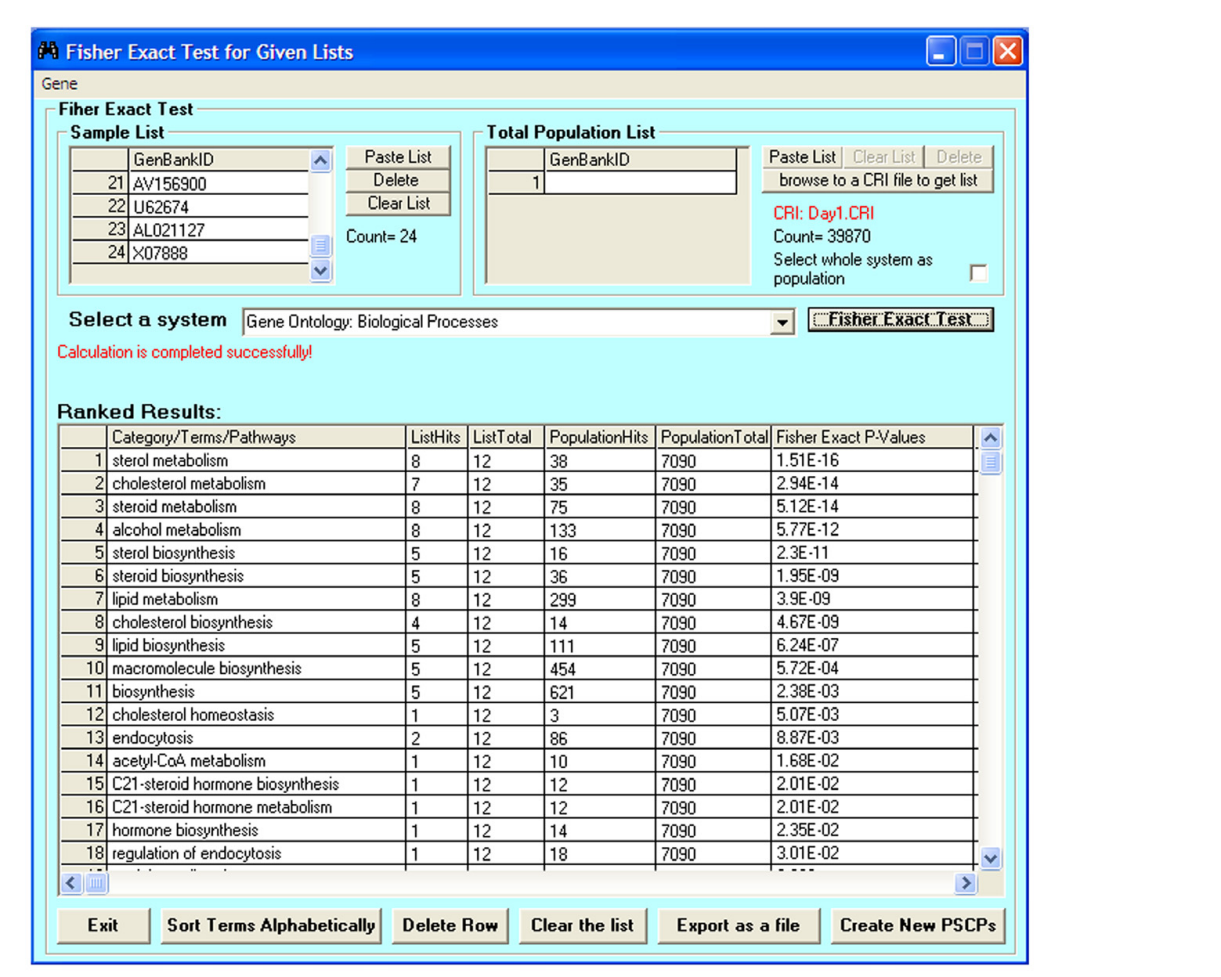
The <Fisher's exact test for Given Lists> window. The gene list derived from pattern extraction in Fig. 6 was copied and pasted into this window. The gene list from Day 1 CRI file was chosen as the total population list. GO: Biological Process was used as the system for Fisher's exact test. The computation results are ranked based on the p-values of each category (i.e. GO term) of the system. The less p-value, the higher the category ranks.

One strategy to analyze microarray or other HTP data is to look for genes with certain user-defined expression patterns across one or more datasets with some biological implications and themes. The expression level-switch phenomenon at birth and weaning across the time course experiment within the cholesterol synthesis pathway described earlier (Fig. 5), prompts us to further investigate the underlying mechanisms and relevant genes and biological processes to cholesterol metabolism. We used WPS to perform pattern extraction with a defined color criteria pattern that reflects the expression level switch around the birth and weaning time points from the time course experiments (Fig. 6). The pattern-matching panel and color template affords the user great flexibility in determining which dataset(s) to include and what color(s) to accept (Fig. 6). The resulting gene list containing 24 unique genes was copied and pasted to Fisher's exact test window to evaluate the statistical enrichment of pathways or GO terms within this list (Fig. 7; also see Additional file 8 for complete result list). As expected, the Fisher's exact test clearly indicated enrichment of sterol/lipid metabolism and synthesis GO terms in the resulting gene list (Fig. 7).

### Gene-Term Association Network (GTAN) for gene-specific functional subnetwork domains or function-oriented gene clusters

**Figure 8:**
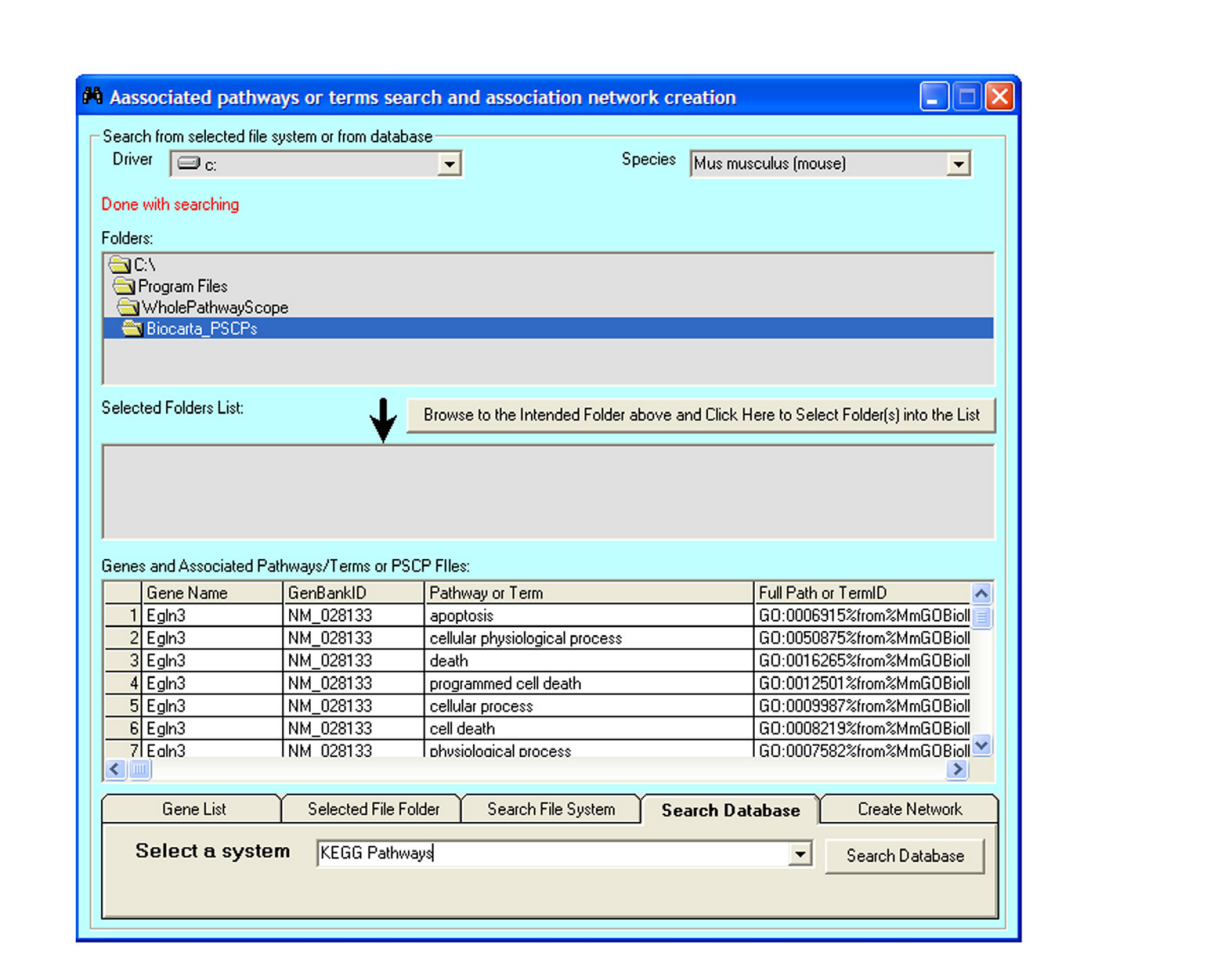
The window for generation of gene-term association network. The gene list derived from pattern extraction in Fig. 6 was copied and pasted into this window. GO:Biological Process, Biocarta, and KEGG pathways were searched with the gene list and results were entered into the table in a gene-term pairwise format.

**Figure 9:**
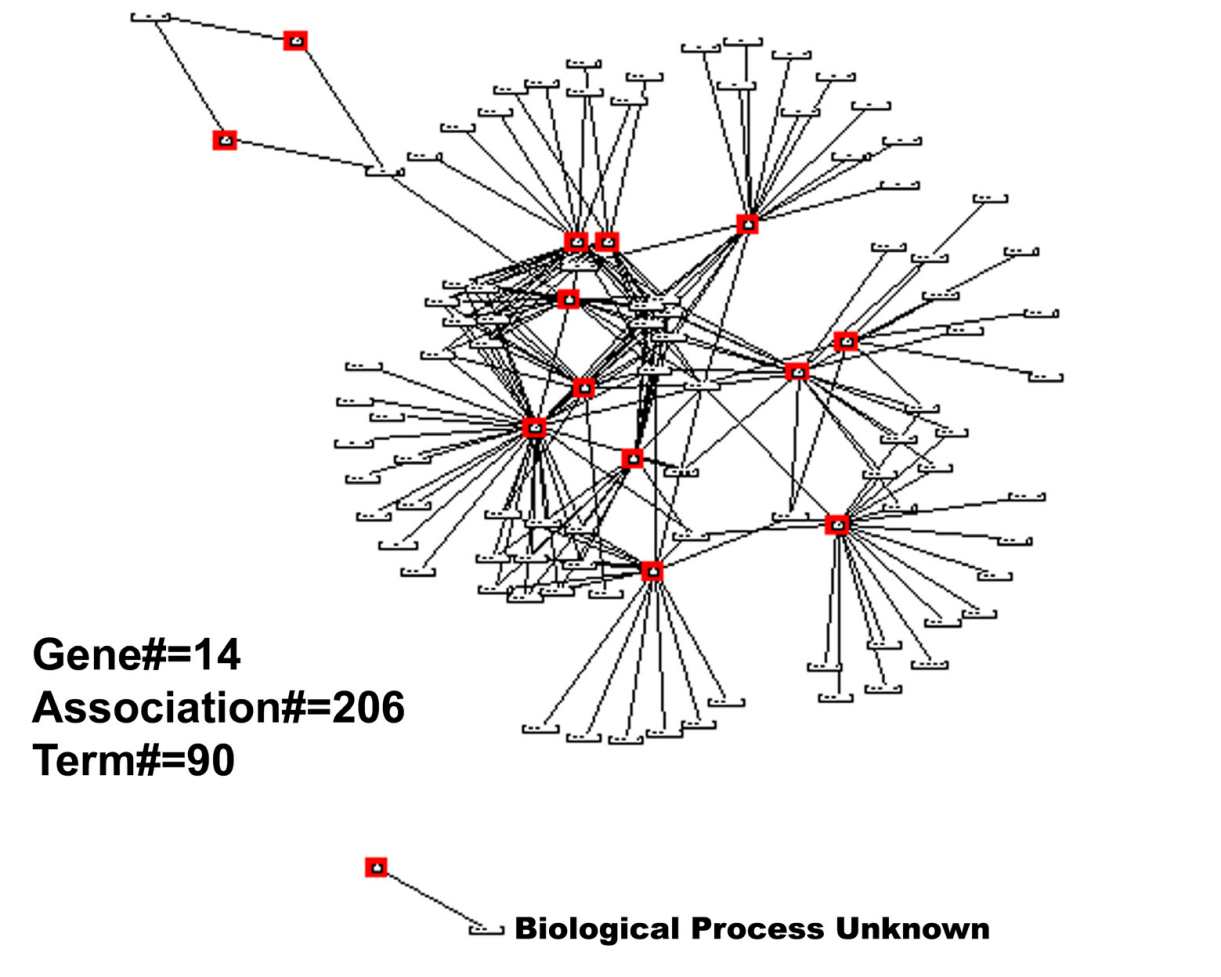
Graphical display of a gene-term association network (GTAN) within WPS. A GTAN was created based on the searched result from Fig. 8. The numbers of gene, association, and terms in this GTAN are indicated. All the genes in the network are highlighted in red. The rest of the tags in the network are pathway tags representing pathways/terms. The lines linked the genes and associated terms indicated the associations (Note: This diagram is to show the whole layout of the network. When running WPS, the legends of genes and pathway tags can be visibly seen when mouse is placed on top of the tags even if they are not visible in this diagram.)

**Figure 10:**
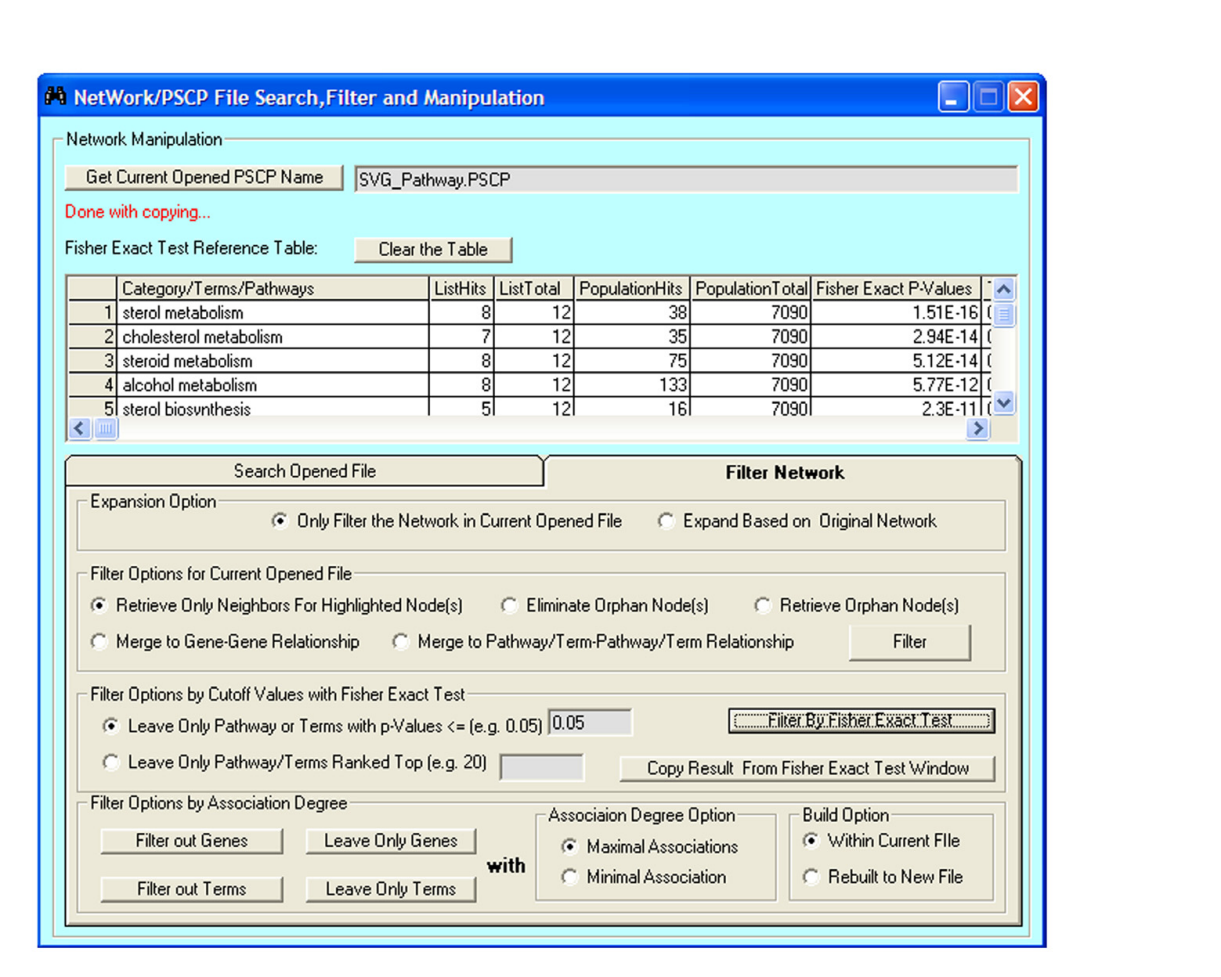
Window for GTAN filtering and manipulation. Many filtering options are available including Filtering by Fisher's exact test result, by association degree, and by retrieving neighbors for highlighted nodes, merging to gene-gene or term-term network, and disease-associated gene highlight feature.

To further study the underlying relationships between genes and involved/enriched pathways or GO terms, we used WPS to search involved pathways from Biocarta, KEGG and GO/Biological Processes terms for the extracted gene list from Fig. 6, and then dynamically generated a gene-term association network (GTAN) (Fig. 8, 9). Thereby, we visualized gene-term association as well as gene-gene and term-term relationship in a graphical manner. Within a typical GTAN, any gene with an associated term has a line linked to it representative of the association relation. In this interactive window, the line linkage between the gene and its associated term will still be maintained even if the gene or the term is moved to a different location. As shown in Fig. 9, among total 24 genes from the above extracted gene list, only 14 genes have annotated association pathway/GO terms from Biocarta, KEGG and GO/Biological Processes, totalling 90 terms and 206 gene-term associations, which are included in the network (see Additional file 12 for complete pair-wise gene-term relations in this GTAN). All the genes that are highlighted in red (Fig. 9) are presented as a network along with their associated pathways/terms. At this level of a GTAN, the legends of genes and associated terms are invisible (Fig. 9). One can explore the network by moving the mouse over the top of genes or terms, the legend of which can then be displayed visibly near the mouse. Alternatively, one can "zoom in" on a selected area of the network to take a closer look at the subnetwork. There is also a specialized window available for users to manipulate, filter, and explore the network (Fig. 10).

**Figure 11:**
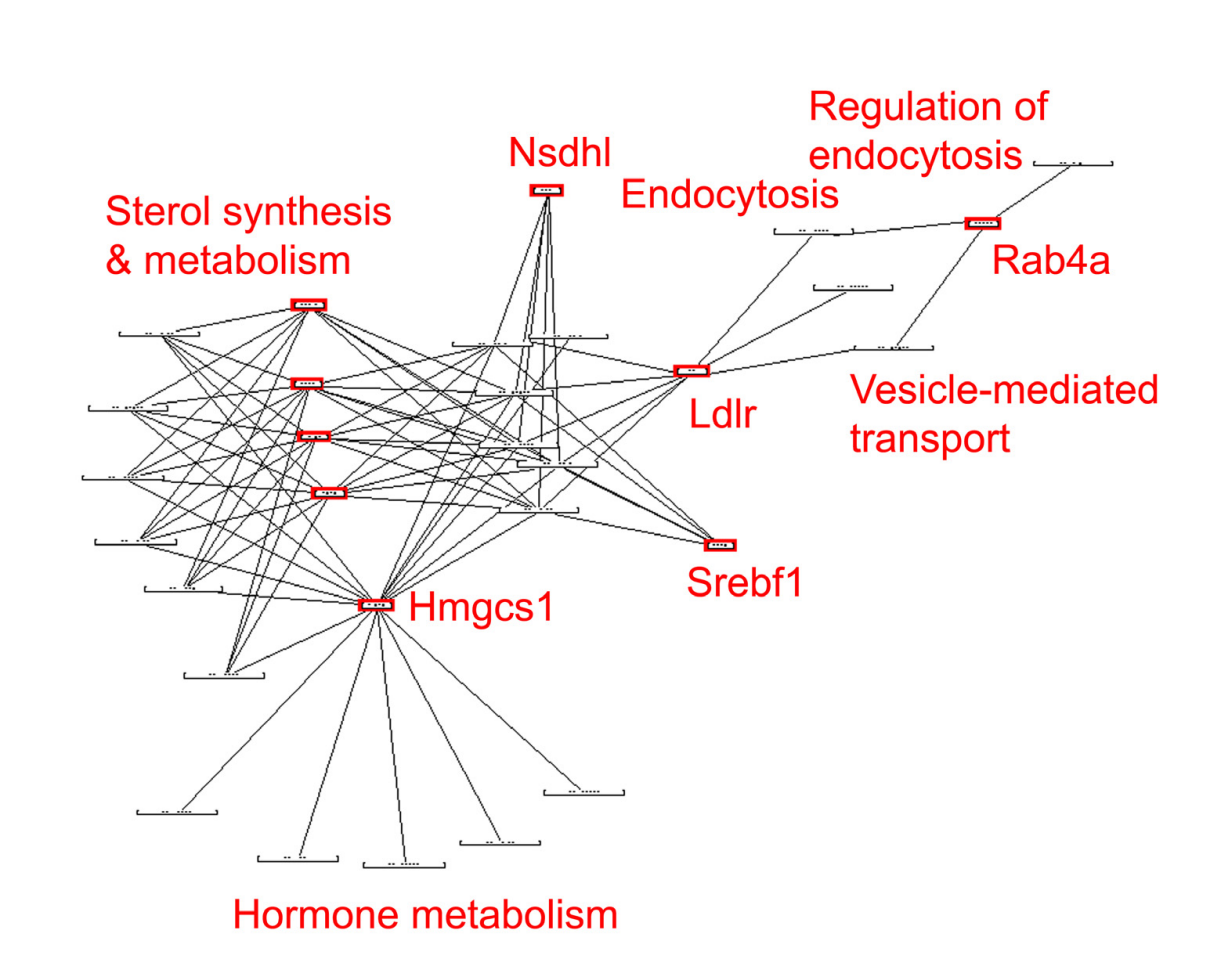
Manipulation and filtering of GTAN to gain insights of overall relationships. The GTAN from Fig. 9 was filtered using the Window from Fig. 10 based on Fisher's exact test result shown in Fig. 7 as reference using cutoff p-value < = 0.05. Genes are highlighted in red. All the terms shown in the network have p-values < = 0.05, some of which are labeled individually and some of which are labeled as summarized descriptions due to close relevancy.

To investigate the biological themes from the resulting GTAN, we used a variety of manipulation methods available in a utility window to filter and simplify the network within WPS (Fig. 10). In combination with the Fisher's exact test result (Fig. 7), we filtered out a subnetwork of genes with associated GO terms that have Fisher's exact test p-values no larger than 0.05 (Fig. 10, 11). Interestingly, although the majority of the enriched terms are engaged in sterol/lipid synthesis and metabolism, endocytosis and vesicle-mediated transport GO terms, which are enriched in the extracted gene list, link *Ldlr *and *Rab4a *together in the subnetwork (Fig. 11). We also retrieved terms with minimal associations with genes, which tend to be unique terms/pathways describing their involved genes (see Additional file 13). Notably, *Rab4a *is functionally involved in signaling and cell communication (see Additional file 13). Thus, it appears that *Rab4a *could be the critical signaling component that triggers and mediates the sterol/lipid synthesis machinery in the body through *Ldlr *, probably by the mechanism of endocytosis (Fig. 11, and see Additional file 13).

### Shared term extraction and disease gene annotation

**Figure 12:**
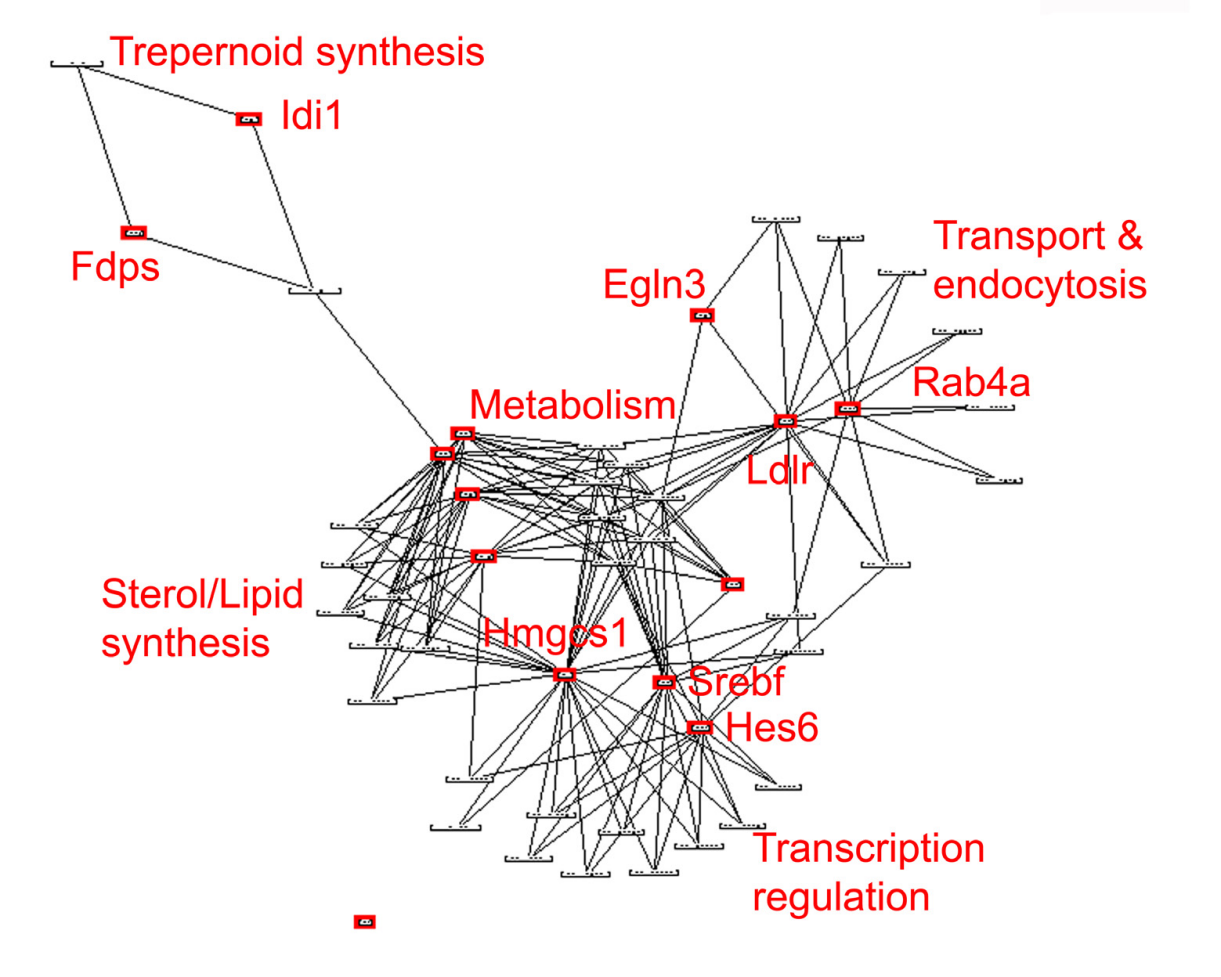
Manipulation and filtering of GTAN based on association degree. The GTAN from Fig. 9 was filtered to get rid of terms with minimal association of genes. Genes are highlighted in red. Terms tend to be shared by multiple genes and are labeled as summarized descriptions for simplicity purpose.

We then filtered out genes with minimal association of terms and looked for shared terms within the network (Fig. 12). As evident in Fig. 12, sterol/lipid synthesis and metabolism are major shared terms in the network, which is consistent with the filtered network from the Fisher's exact test result (Fig. 11). The fact that *Hmgcs1 *, *Hes6 *and *srebf1 *(SREBP) heavily shared transcription-related terms (Fig. 12), is consistent with the facts that *Hmgcs1 *and *srebf1 *have been previously shown to be involved in sterol metabolism regulation and *Hes6 *has been implicated in transcriptional regulation [48], suggesting that they may collaborate in metabolism-related transcriptional regulation. *Egln3 *only shared high level or more generic GO terms (e.g. cellular biological process) with other genes (Fig. 12), confirming its unique functional involvement in cell death among the gene list (see Additional file 13). Furthermore, *Rab4a *and *Ldlr *shared many pathway/terms, suggesting these two may be functionally coupled.

Interestingly, when we used disease-association highlight feature in WPS (see Additional file 6 for screenshot), we found that *Rab4a *, *Ldlr *, and *srebf1 *are all more or less associated with obesity annotated in Genetic Association Database [33] and MedGene databases [34] (data not shown).

### Gene-gene networks and neighbor identification

To study how genes are related to each other directly within the network, we merged the gene-term association network in Figure 12 into a gene-gene network by their shared terms, after eliminating some generic terms for clarity purposes. The gene-gene network formed a domain-like architecture (see Additional file 14, Fig 13). The genes that are heavily involved in sterol/lipid synthesis and metabolism formed a clustered domain with massive links among them. Distinct genes with different functional trends from others tended to be separated out of the "crowded" region such as *Egln3 *, *Hes6 *, and *Rab4a *.

**Figure 13:**
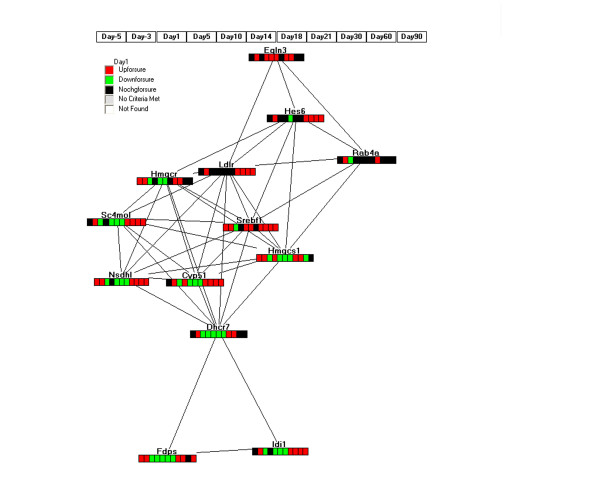
Manipulation and filtering of GTAN to gain insights of gene-gene relationships. The GTAN from Fig. 12 was merged into a gene-gene network through shared terms: if two genes share a common term, then draw a line in between these two genes and get rid of the term and its original association lines, and then the graph was rebuilt by SVG (see Additional file 14). Before merging, some generic GO terms (high rank in GO hierarchy, e.g. physiological process) were eliminated for simplicity purpose. Genes are selected by highlighting in red for further manipulation (see Additional file 14). Then the obtained PSCP file containing the merged network was colored by the CRI files from the time course experiments (same CRI files as in Fig. 5).

**Figure 14:**
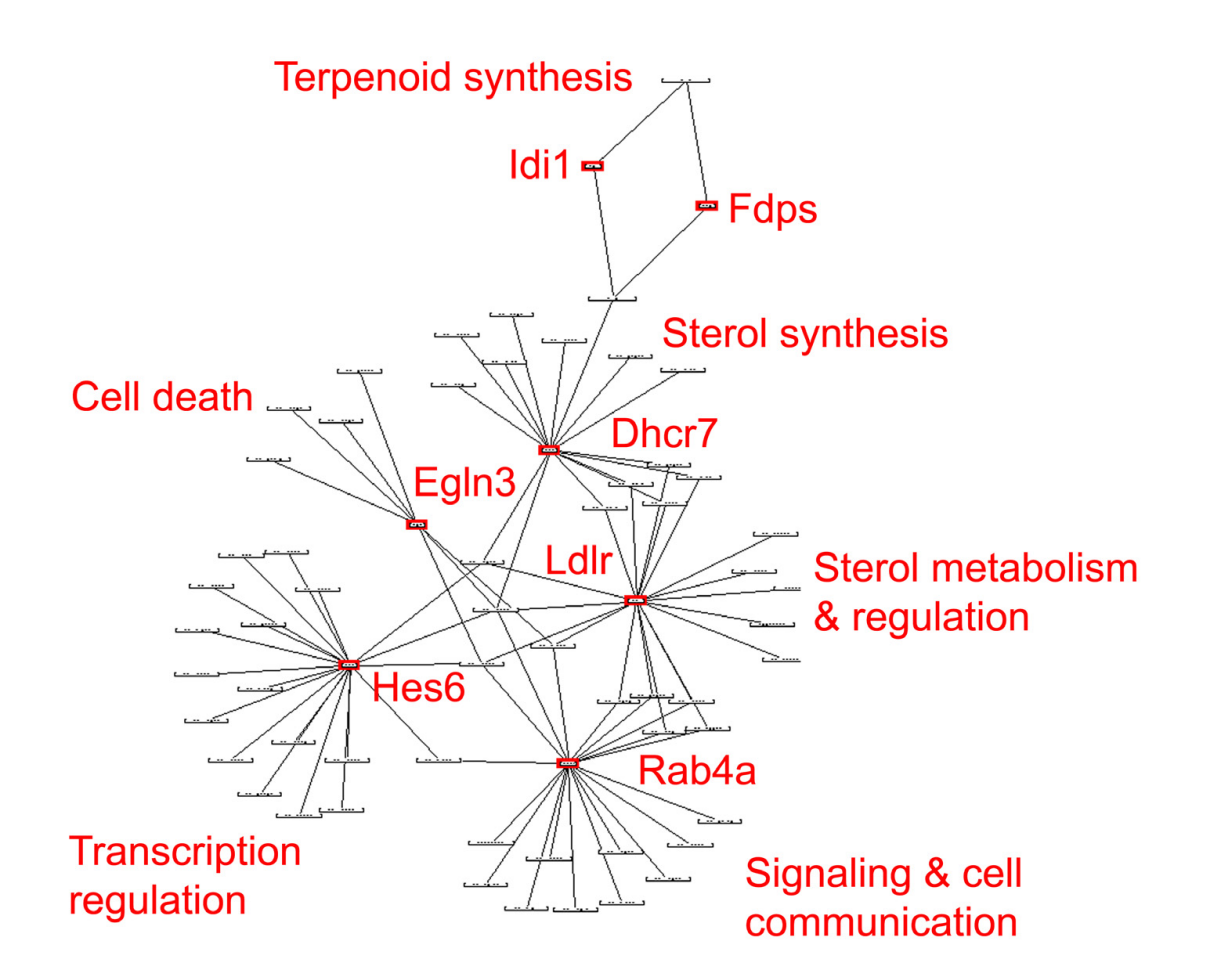
Manipulation and filtering of GTAN to gain insights of gene-term relationships. Highlighted the selected genes in Fig. 13 including Egln3, Hes6, Ldlr, Rab4a, Dhcr7, Fdps, and Idi1, and then retrieved their neighbors (terms) to re-build the graph by SVG. Terms are labeled as summarized descriptions due to close relevancy.

In order to learn more relationships among these "distinct genes", we highlighted them in red with WPS (see Additional file 14, Fig. 13) and then retrieved the immediate neighbors for them from the original GTAN network to create a subnetwork (Fig. 14). Since *Ldlr *is the only one from the "Sterol metabolism clustered domain" that connects to *Egln3 *, we also included it for further analysis. Notably, within the newly created subnetwork, all the selected genes are linked, although distinct domains or clusters for each gene are apparently separated very well (Fig. 14). All these domains may represent the major functional aspects underlying the "expression level-switch" phenomenon and are networked together as a dynamic biological theme. Interestingly, these genes were originally selected due to a similar expression pattern across the "cholesterol metabolism" switch points (Fig. 6, Fig. 13). Especially, *Rab4a *, *Egln3 *and *Hes6 *have been previously implicated in their functional categories including signaling and endocytosis [49], cell death [50] and transcription regulation [48], respectively. Whether and/or how they play roles in cholesterol metabolism related signaling, cell death and transcription regulation remains to be investigated.

## Discussion

### WPS has unique features not found in a single similar application

The new software program, WPS, is described that facilitates and enhances the analysis of HTP data. Unique features of WPS include the ability to simultaneously display HTP data from multiple experiments within the context of known biological pathways, visualizing and analyzing gene-pathway/term and gene-gene relationships and biological implications within created gene-term association networks, extracting a gene list that may reflect certain biological themes by means of a user-defined pattern template with color cues, and statistically estimating the enrichment of biological pathways or GO terms within a distinguished list or a PSCP file under analysis (see Table 2 for comparison with several free and commercial pathway analysis tools).

WPS also interfaces easily with clustering programs by accepting the gene lists from clustering analysis. This will aid in the identification of interactions among biological pathways and relating the expression profiles of genes of unknown function to those of established pathways. The program accepts data from any microarray platform (including oligonucletide arrays and cDNA arrays), and accommodates data generated by SAGE (serial analysis of gene expression) [51] as well as proteomics data.

In summary, WPS was developed to provide the following important features which were not previously available under a single application (Table 2):

### Analyze multiple datasets simultaneously

First, many of the current programs display data from just a single HTP experiment. This limitation hampers direct visual comparison of results from different HTP experiments. When the number of datasets is large, as in a typical time course experiment, it becomes much harder for investigators to remember what is happening at each time point. So far, to our knowledge, our unique way in WPS of displaying multiple datasets simultaneously is absent from most, if not all, of the free and commercial pathway-based HTP data analysis tools. Even a very large number of datasets, if pre-processed and combined for same or similar categories, can still be effectively displayed and visualized in the program. In our example, shown in Figure 5, the ability of WPS to display and analyze datasets of a time course experiment in the cholesterol synthesis pathway simultaneously is very helpful for pattern recognition, especially within the genes in this pathway at the time points of birth and weaning (Most genes tended to be down-regulated after birth and up-regulated after weaning). This is more difficult to discover in such an easy and intuitive way within a pathway without using the simultaneous coloring feature of the program. Benefited from this feature in WPS, the resulting pattern recognition in turn would lead to pattern extraction of a specific color pattern in WPS, which might have biological implications (Fig. 6, discussed below).

### Analyze multiple pathways simultaneously and generation of GTAN to explore gene-term relations in an intuitive graphical manner

The second feature that distinguishes WPS from current microarray programs is the ability to display multiple pathways simultaneously, either in their entirety or in summary form. A given collection of pathway files can be grouped into a single WSCP file by means of pathway tags, and changes in the behavior of genes in each pathway can be flagged according to criteria specified by the user. Thus, the pathway(s) that are significantly affected by the experimental conditions are easily identified without having to visualize each pathway individually.

Furthermore, the generation and manipulation of biological gene-term association network (GTAN) greatly expands the capacity to study the gene-term and gene-gene relationships in a genome-wide fashion and provides a new way to look at genes and their involved pathways or functional GO terms. WPS has the statistical capacity, specifically using the Fisher's exact test method, to identify over-represented biological themes (pathways/processes/GO terms) in a given list of genes. More importantly, the filtering of the GTANs based on the Fisher's exact test result would give rise to a subnetwork enriched in genes and terms/pathways with statistical significance. This would help to narrow down the "core" genes and their associated terms/pathways with biological relevance of higher priority. A solid example of network filtering with the help of Fisher's exact test result is described in Fig. 11. Endocytosis and vesicle-mediated transport, which are enriched in the extracted gene list, co-exists and connects with the major enriched function sterol/lipid synthesis and metabolism in the filtered subnetwork. The layout of the filtered subnetwork brought these functions together with visible relations within the genes of interest (Fig. 11). Some specialized tools with more sophisticated statistical methods have been previously described including EASE [44,52], Fatigo [53] and GOMiner [54], which prioritize biological themes embedded in the given gene list. However, none of these tools directly visualize and study the gene-term and gene-gene relationship within the biological contexts represented by the gene list in an intuitive graphical manner, nor do they take advantage of the statistical enrichment of terms to grasp the relations of the key genes and their associated terms/pathways like in WPS. In contrast, within a GTAN created by WPS, displaying all possible associated pathways/terms for genes of interests and allowing the dynamic layout of a network based on the current biological contexts, will allow one or more context-dependent, specific functional terms to be assigned to the gene based on its current role in such context. On the other hand, its shared terms with other genes would implicate the biological connection of this gene with its "neighbor" genes. We believe such visual cues derived from GTANs not only provide an overall biological picture of the current biological context, but also shed light on the function of a gene in the network, which might only be obvious when seen with its partners.

Currently, prediction and creation of genome-wide pathways [55], as well as utilization, and exploration of biological networks (genetic, regulation, and biochemical) as a method for data analysis is becoming a major trend in systems biology and computational biology [56,57]. There are many new tools and algorithms being developed to move in this direction [58-61]. Many use complicated algorithms such as Bayesian network, Petri nets, probabilistic graphical model or newly defined rules to simulate regulatory networks and dynamic trajectories of genes. In addition, most of the commercial tools use gene-gene relationships as their major components of the networks, such that the users may easily lose track of the role of genes. The GTAN approach in WPS is not only simple to use, but also unique and effective in that gene-term association relationships are the major components of the network, so that users can easily keep track of genes and their involved functional terms or pathways and predict gene-gene relationships through their shared terms.

### Color cue template-based pattern extraction of gene lists for biological themes

A third unique feature of WPS is pattern extraction, which is different from other pattern or profile-based approaches (e.g. typical clustering and classification methods) and some statistics-based methods (e.g. SAM [18]). Instead of relying solely on data values, the pattern extraction method in WPS takes advantage of user-defined color criteria in CRI files representing HTP datasets. Since users define criteria in CRI files with logical expression and not just based on the data values of genes in datasets, genes with quite different data values can be defined as the same class of data in terms of their behaviors. For example, a gene with fold change of 2 may be defined into the same data class category as another gene with fold change of 8, if the user defines "no less than 2 fold" as up-regulated genes. If the user defined this criterion with red color, then red color would represent a class category of genes, which are up-regulated, with fold change no less than 2 fold no matter whether it is 2 fold or 8 fold change, as long as the user is confident with the definition based on his own experience. In fact, one can enhance this definition by adding other quality control factors such as p-value in the definition logical expression, another advantage of CRI color criteria. Thus, this kind of definition eliminates the mathematical difference but maintains the embedded biological meanings in the data values, since biological processes are more qualitative than quantitative in most cases.

### Limitations and future direction

WPS facilitates comprehensive analysis and visualization of HTP data within the context of known biological pathways and gene-term association networks. The program will continue to be improved, as characterization of biological pathways and networks becomes increasingly comprehensive and challenging. The ultimate goal of WPS is to integrate all the available information and databases as well as an individual user's data with different forms and formats in the contexts of biological pathways and networks.

The current version of WPS is a windows-based program and serves as proof of concept of pathway/gene analysis of HTP data. The future version of the program will move to a three-tier architecture in a production-scale platform to allow WPS, through a middle layer, such as Java Servlets, to communicate with the server's resource, which may have excellent data storage as well as computation capacity. Its front end interface will also evolve into a platform-independent client such as a web-browser, depending on resource performance and other factors. Integration with other data sources and additional pathways are also to be added, so that the magnitude of HTP data analysis can be largely extended with the power of an expandable server.

WPS provides Fisher's exact test method for statistical evaluation in both global system and local current PSCP files either derived from internal database or user-customized under analysis. It could be improved by the addition of more sophisticated statistical utilities such as false discovery rate (FDR) estimation [62] or other statistical enhancements (e.g. bootstrap method) to analyze HTP data and determine the significance of functional enrichment in individual pathways or GO terms in a more solid way. Computational requirements limit the full integration of these statistical methods, but even without them, our software contributes significantly to improve integrative data analysis.

## Conclusion

We have described WPS, as a new pathway-based analysis tool, that facilitates and enhances the analysis of HTP data in the context of biological pathways and networks. WPS has many unique features not found in a single existing application. WPS has implemented a clustering analysis-like approach but using a more biologically relevant approach in the color cue-templated pattern extraction method. In addition, WPS uses Fisher's exact test to evaluate statistical significance of identified genes. Finally, WPS incorporates pathway and association network-based biological contexts as a platform, and unique coloring scheme with multiple datasets and multiple pathways as an intuitive way to visualize and analyze data of different resources. This is likely to be important for comparison of HTP data from diverse sources such as microarray and proteomics. Within WPS, the new way of pattern extraction may provide another dimension for uncovering genes with more quality-based, not just quantity-based, expression patterns likely with implications and themes more closely related to ongoing biological processes. Within WPS, the new way of visualizing and analyzing the biological relations among genes, pathways, and terms under GTANs provides a new platform for integrated discovery. This tool represents a pathway-based platform for discovery integration to maximize analysis power.

## Availability and requirements

**Project name**: Pathway analysis tool WPS for high-throughput data;

**Project home page**: [63]

**Operating system**: Microsoft Window 2000 or XP

**Programming language**: Microsoft Visual Basic 6

**Other requirements**: Internal databases for different species and a collection of over 1900 PathwayScopeFiles (PSCP files for mouse) available on web site; Additional user-provided PSCP files and those from other sources will be made available as they are collected.

**License**: Free to academics; distributed through license agreement

**Any restrictions to use by non-academics**: commercial license needed

## List of abbreviations used

WPS – WholePathwayScope

PSCP – PathwayScope File

WSCP – WholeScope File

CRI – Criteria File

GTAN – Gene-Term Association Network

HTP – High Throughput

G5G8 – *ABCG5 *and *ABCG8*

ABC – ATP-binding cassette

Tg – Transgenic

KO – Knockout

KEGG – Kyoto Encyclopedia of Genes and Genomes

CGAP – The Cancer Genomes Anatomy Project

SVG – Scalable Vector Graphics technology

GO – Gene Ontology

SAGE – Serial analysis of gene expression

## Authors' contributions

**M Y **– Programmer Analyst III and Scientific Application Specialist from ABCC, responsible for design and implementation of WPS, a former member of UTSW.

**J D H **– Assistant Professor of UTSW, a collaborator who provided microarray data in the application examples.

**J C C **– Associate Professor of UTSW, a scientific partner of H H H, responsible for initial design of WPS in UTSW.

**H H H **– Director of McDermott Center and Investigator of HHMI, responsible for initial funding and design of WPS in UTSW.

**R M S **– Senior author, Supervisor of M Y in ABCC, responsible for design and improvement of WPS and funding for WPS in ABCC.

## Supplementary Material

Additional File 1 A Microsoft PowerPoint file including a few slides of screenshots to describe the features for displaying a Biocarta pathway graph and highlighting selected genes to display their data in the graph. **Slide1: **The window for creating a PSCP file for a Biocarta pathway from the internal database. **Slide 2: **The PSCP file including all the genes in the created Biocarta pathway "FXR and LXR Regulation of Cholesterol Metabolism". **Slide 3: **Color the created PSCP file with loaded CRI files (the time-course data used in Fig. 5) with gradient coloring scheme. **Slide 4: **WPS can display the corresponding Biocarta pathway diagram in a separate internet browser and show the data with designated arrows (red arrows) for the selected genes highlighted in created PSCP file (slide 3). Clickable buttons labeled with names of loaded datasets are to allow displaying of data for corresponding CRI file for the selected genes. Click here for file

Additional File 2 A Microsoft PowerPoint file including a slide of a screenshot for a microarray raw dataset in a worksheet of an Excel file to graphically illustrate the format and 3 requirements of a data file to be converted into a CRI file in WPS. Click here for file

Additional File 3 A Microsoft Word file including a detailed description of the three types of files in WPS. Click here for file

Additional File 4 A Microsoft PowerPoint file including a few slides of screenshots to describe the feature for pattern extraction of genes from a colored PSCP file. **Slide1: **A colored PSCP file (previously has been loaded with CRI files) subjected to pattern extraction. **Slide 2: **The pattern extraction window for extraction of genes from the colored PSCP file in slide 1 that match with the defined color pattern in the color template panel. **Slide 3: **The created PSCP file including the extracted genes in slide 2 to verify the pattern of extracted genes colored with same set of CRI files. Click here for file

Additional File 5 A Microsoft Word file including description of the feature for manipulation and filtering of GTANs. Click here for file

Additional File 6 A Microsoft PowerPoint file including a slide for screenshot of the window for searching network for specific genes or terms or for disease-associated genes. The selected disease from database is used to search and highlight the associated genes in current GTAN/PSCP file for further analysis. Click here for file

Additional File 7 A Microsoft PowerPoint file including a slide for illustration of a 2 × 2 contingency table used as basis for Fisher's exact test. Click here for file

Additional File 8 A Microsoft Excel file including an example result of Fisher's exact test exported from WPS in Figure 7. Click here for file

Additional File 9 A Microsoft PowerPoint file including a few slides of screenshots to describe the feature for pathway or PSCP-scoped "local Fisher's exact test" of user-defined pattern enrichment of choice genes colored with CRI file(s) in a PSCP file being analyzed. **Slide1: **A colored PSCP file (previously has been loaded with CRI files) subjected to "local Fisher's exact test". **Slide 2: **The "local Fisher's exact test" window for measuring statistically the enrichment of genes with user-defined criteria, in this example, the enrichment degree of differentiated expressed genes (red and green colors in the color template panel) for each dataset within this pathway. Click here for file

Additional File 10 A Microsoft PowerPoint file including a slide for screenshot of the window from WPS for sorting a dataset to pathway/term scoped sub-datasets for further processing. Click here for file

Additional File 11 A Microsoft Word file describing the materials and methods for preparation of microarray data used for describing the program features. Click here for file

Additional File 12 A Microsoft Excel file including the complete pair-wise gene-term relations in the GTAN in Fig. 9. Click here for file

Additional File 13 A graphical tif file to illustrate a filtered GTAN from the GTAN of Fig. 9 for terms with minimal associations of genes, which tend to be unique or specific for their associated genes. Genes are highlighted in red. Click here for file

Additional File 14 A graphical tif file to illustrate a GTAN derived from the GTAN of Fig. 12 by merging into a gene-gene network through shared terms. Some genes are highlighted in red used for further manipulation. Click here for file

Additional File 15 A Shockwave Flash file to show a movie clip as a program demo for how to convert a dataset file to a CRI file to be used in WPS. (Note: the movie files can be viewed directly using internet browser with Flash Animation plug-in) Click here for file

Additional File 16 A Shockwave Flash file to show a movie clip as a program demo for how to load CRI files(s) to color a PSCP or a WSCP file. Click here for file

Additional File 17 A Shockwave Flash file to show a movie clip as a program demo for how to create a PSCP file or WSCP file from the internal database. Click here for file

Additional File 18 A Shockwave Flash file to show a movie clip as a program demo for how to do pattern extraction from selected CRI file(s), how to do the global Fisher's exact test for a given list (e.g. a gene list from pattern extraction), and how to create a GTAN from a given list. Click here for file

Additional Files 19–34 zip files that include each of 16 microarray CRI files used in the application examples in the manuscript (Day-5.zip, Day-3.zip, Day1.zip, Day5.zip, Day10.zip, Day14.zip, Day18.zip, Day21.zip, Day30.zip, Day60.zip, Day90.zip, G5G8KO1.zip, G5G8KO2.zip, G5G8KO3.zip, G5G8Tg1.zip, G5G8Tg2.zip) (unzip them using WinZip program or other appropriate programs before use). Click here for file

Additional Files 35–50 zip files that include each of 16 microarray raw files (Excel files) used in the application examples in the manuscript (rDay-5.zip, rDay-3.zip, rDay1.zip, rDay5.zip, rDay10.zip, rDay14.zip, rDay18.zip, rDay21.zip, rDay30.zip, rDay60.zip, rDay90.zip, rG5G8KO1.zip, rG5G8KO2.zip, rG5G8KO3.zip, rG5G8Tg1.zip, rG5G8Tg2.zip (unzip them using WinZip program or other appropriate programs before use) Click here for file

## Figures and Tables

**Table 1:** An example of gene criteria for the microarray dataset for *G5G8 *^*Tg *^mice vs wild-type mice.

**Criteria Priority**	**Criteria Name**	**Criteria Details**	**Color**
**1**	**UpHighCertainty**	([TGvsWT_A_Change] = 'I' OR [TGvsWT_A_Change] = 'MI')AND ([WT_A_Detection] <> 'A' OR [TGvsWT_A_Detection] <> 'A')	**red**
**2**	**DownHighCertainty**	[TGvsWT_A_Change] = 'D' OR [TGvsWT_A_Change] = 'MD') AND ([WT_A_Detection] <> 'A' OR [TGvsWT_A_Detection] <> 'A')	**green**
**3**	**UpLowCertainty**	[foldchange] >= 2	**orange**
**4**	**DownLowCertainty**	[foldchange] < = 0.5	**light blue**
**5**	**No Criteria Met**	Program built-in criteria (all of the above criteria not met)	**gray**
**6**	**Not Found**	Gene does not exist in microarray dataset	**white**

[TGvsWT_A_Change]: Change call of Affymetrix array for genes in Transgene array in compared to Wild type array (I: Increase; D: Derease; MI: Medium Increase; MD: Medium Decrease)

[WT_A_Detection]: Detection Call of Affymetrix array for Wild Type array (A: absent, P: present).

[TGvsWT_A_Detection]: Detection Call of Affymetrix array for G5G8Tg array (A: absent; P: present).

**Table 2:** Comparison of major features of WPS with other pathway analysis tools.

	WPS	GenMAPP	Pathway Processor	Cytoscape	PathwayAssist	Ingenuity	MetaCore	PathArt
Customized Pathways or Gene Groups	Yes	Yes	No	?	Yes	Yes	Yes	No
Public Canonical Pathway Collection (e.g. KEGG, BioCarta)	Yes	Yes	Yes	Yes	Yes	Yes	Yes	Yes
Color Code Genes with Datasets	Yes	Yes	Yes	Yes	Yes	Yes	Yes	Yes
Color Code Pathways (Pathway Tags) with Datasets	Yes	No	No	No	No	No	No	No
Concurrent Visualization of Multiple Datasets (in Colored Pathways/Network)	Yes	No	No	No	No	No	Yes	No
Multiple Datasets Visualization Once a Time (in Colored Pathways/Network)	Yes	No	No	No	Yes	Yes	Yes	Yes
Summarized View of Multiple Pathways (i.e. Pathway Tags)	Yes	No	No	No	No	No	No	No
Pattern Extraction from Data Files	Yes	No	No	No	No	No	No	No
Pattern Extraction from Colored Pathways	Yes	No	No	No	No	No	No	No
Global Enrichment Test (e.g. Fisher's exact test) for a Given List	Yes	No	No	No	No	Yes	No	No
Pathway-Scoped Fisher's exact test for Patterned Genes	Yes	Yes	Yes	No	No	No	No	No
Network View (Gene-Gene Association Network*)	Yes	No	No	Yes	Yes	Yes	Yes	Yes
Network View (Gene-Term Association Network*)	Yes	No	No	No	No	No	No	No
Term-based and Fisher's exact test result-based network filtering**	Yes	No	No	No	No	No	No	No
Sort Whole Dataset into Pathway Scoped Sub-datasets***	Yes	No	No	No	No	No	No	No
Types of Products/Availability (Free to Academic: A; Commercial: C)	A	A	A	A	C	C	C	C

WPS has unique features not found in a single similar application. Seven pathway analysis tools (first row of the table: three of them are free to academic and the other four are commercial products) were selected for comparison of major features (first column of the table) with WPS. The other seven tools for comparison include: GenMAPP [27]; Pathway Processor [29]; Cytoscape [28]; PathwayAssist [23]; Ingenuity: Ingenuity Pathway Analysis tool [25]; MetaCore [26]; PathArt [24].

Yes: has the indicated feature; No: does not have the indicated feature at current time;

?: Not clear.

*: Gene-Term Association Network (GTAN); gene-to-term relation binary network in WPS (can be converted to gene-gene association network too); Gene-Gene Association Network: gene-to-gene relation binary network.

**: Filter a GTAN into a more specific subnetwork with terms user selected and highlighted or with ranked terms from Fisher's exact test result (see Fig. 10, 11, 13, 14)

***: Sort a whole dataset into pathway scoped sub-datasets for further analysis: e.g. batch computation of the sub-datasets for pathway-scoped correlation study.
