# Supplementary material for: WholePathwayScope: a comprehensive pathway-based analysis tool for high-throughput data
Source: BMC Bioinformatics. 2006 Jan 19;7:30. doi: 10.1186/1471-2105-7-30 (PMC1388242; doi:10.1186/1471-2105-7-30)

## Slide 1
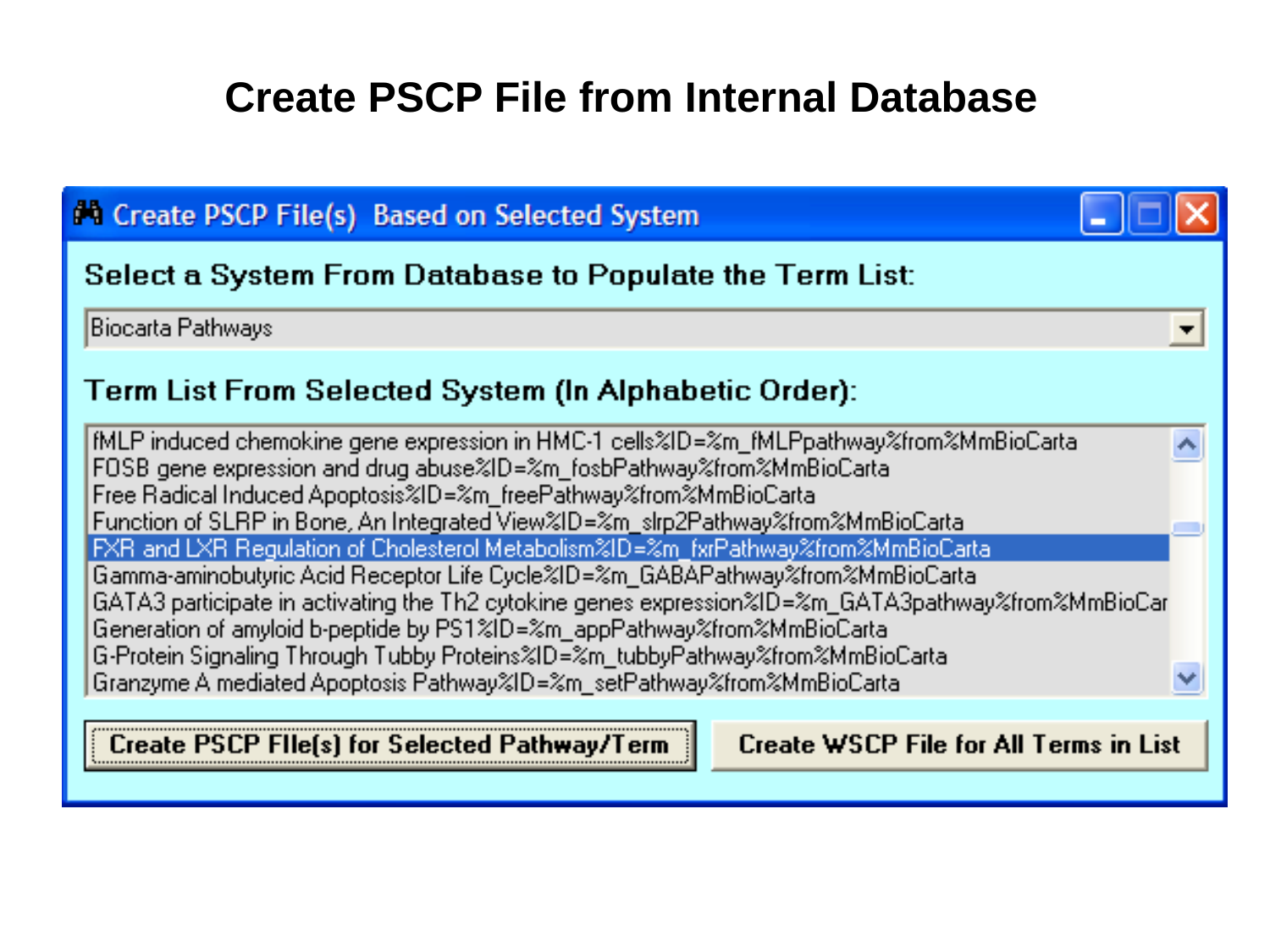

Create PSCP File from Internal Database

## Slide 2
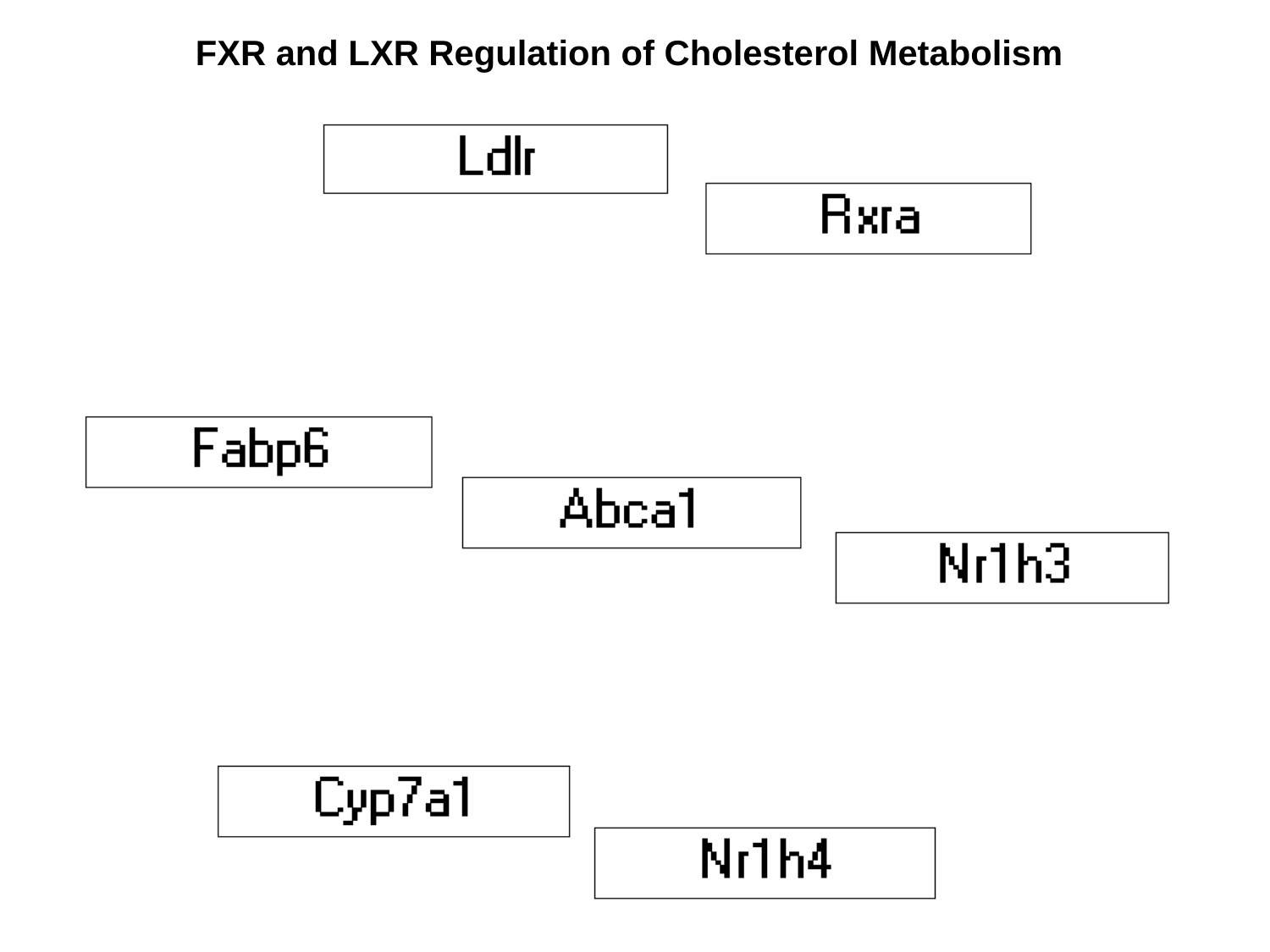

FXR and LXR Regulation of Cholesterol Metabolism

## Slide 3
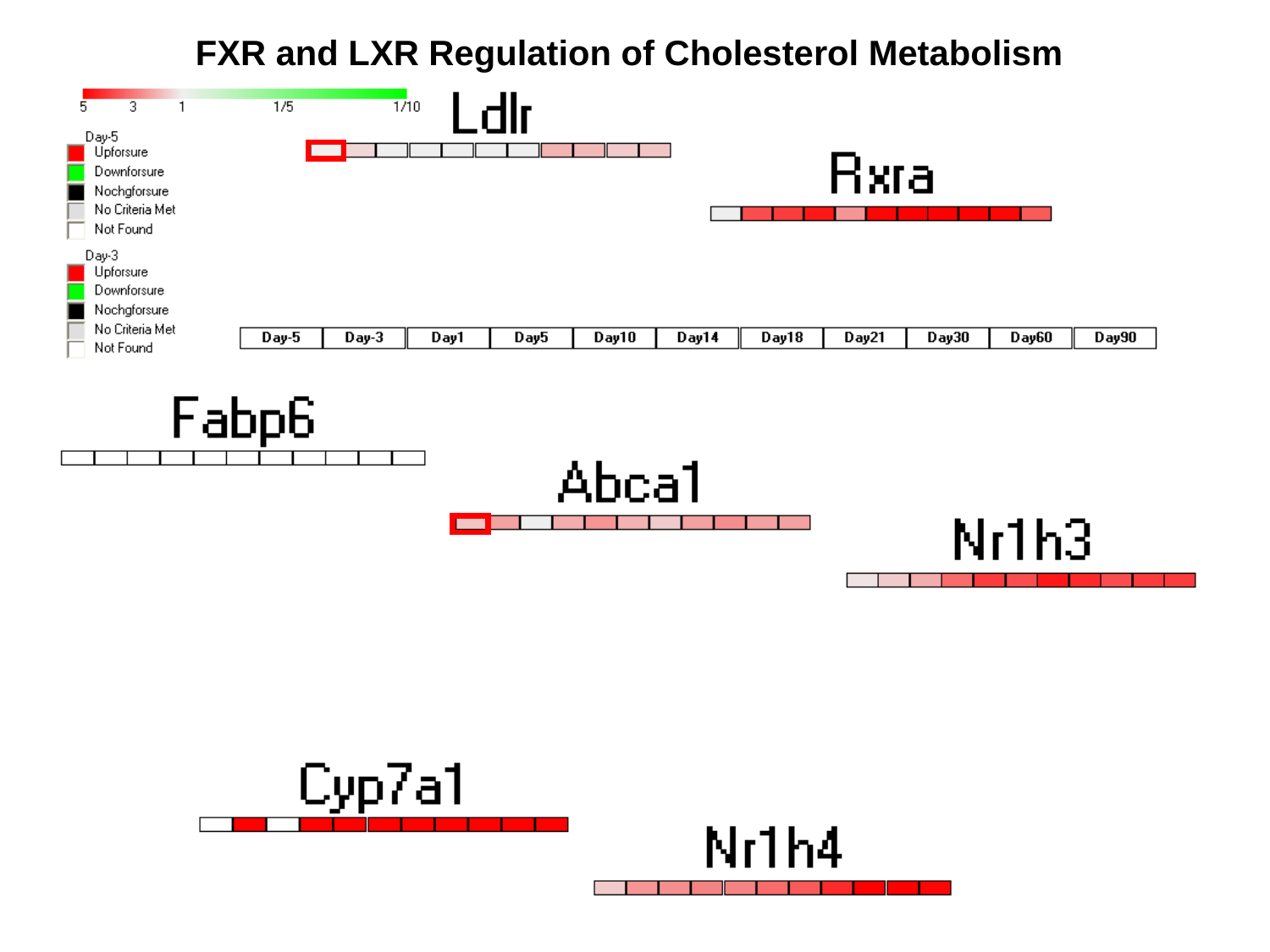

FXR and LXR Regulation of Cholesterol Metabolism

## Slide 4
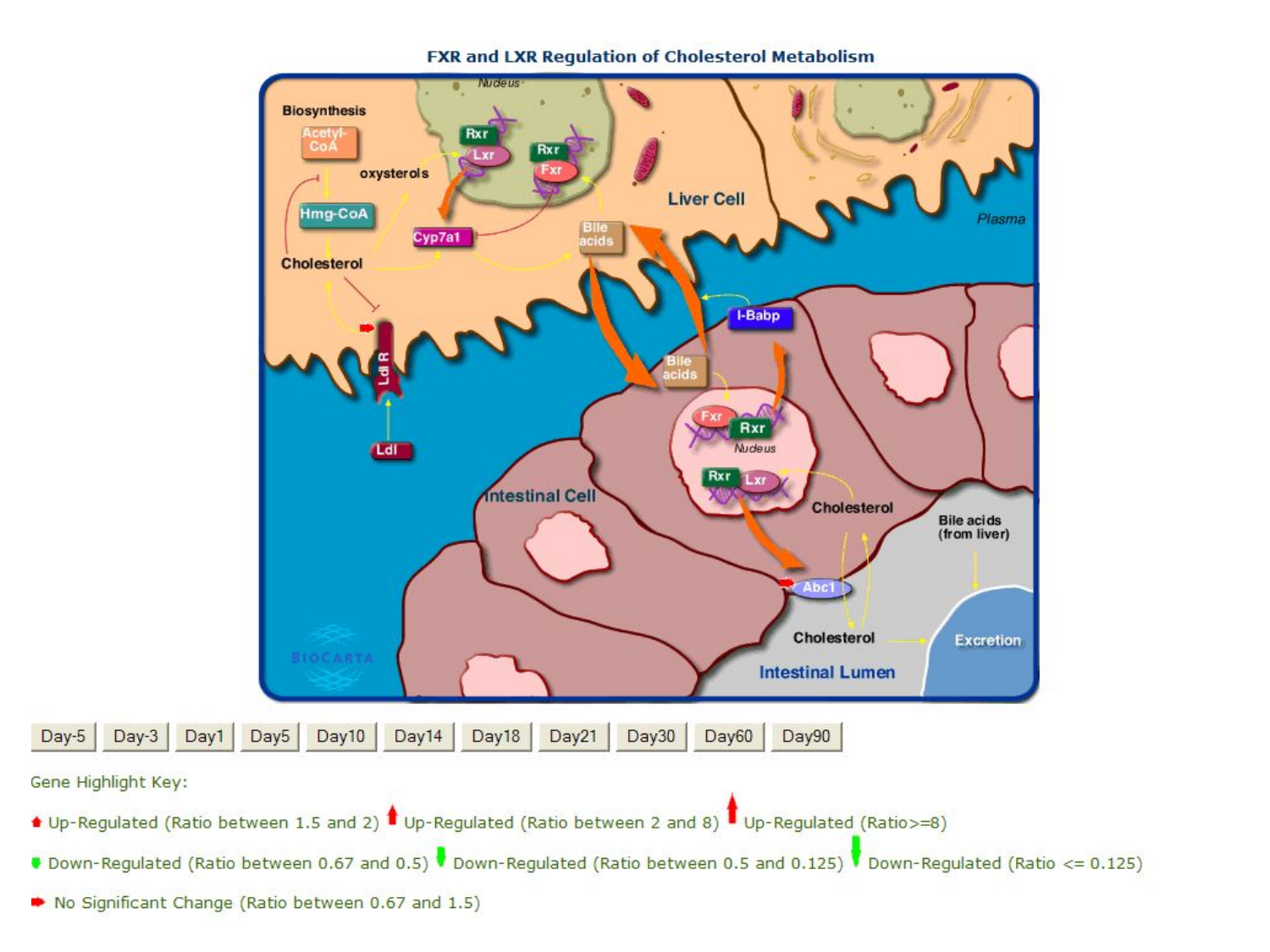

Supplement: Additional File 1 — A Microsoft PowerPoint file including a few slides of screenshots to describe the features for displaying a Biocarta pathway graph and highlighting selected genes to display their data in the graph. Slide1: The window for creating a PSCP file for a Biocarta pathway from the internal database. Slide 2: The PSCP file including all the genes in the created Biocarta pathway "FXR and LXR Regulation of Cholesterol Metabolism". Slide 3: Color the created PSCP file with loaded CRI files (the time-course data used in Fig. 5) with gradient coloring scheme. Slide 4: WPS can display the corresponding Biocarta pathway diagram in a separate internet browser and show the data with designated arrows (red arrows) for the selected genes highlighted in created PSCP file (slide 3). Clickable buttons labeled with names of loaded datasets are to allow displaying of data for corresponding CRI file for the selected genes. [file 1471-2105-7-30-S1.ppt]
