# Supplementary material for: WholePathwayScope: a comprehensive pathway-based analysis tool for high-throughput data
Source: BMC Bioinformatics. 2006 Jan 19;7:30. doi: 10.1186/1471-2105-7-30 (PMC1388242; doi:10.1186/1471-2105-7-30)
Supplement: Additional File 2 — A Microsoft PowerPoint file including a slide of a screenshot for a microarray raw dataset in a worksheet of an Excel file to graphically illustrate the format and 3 requirements of a data file to be converted into a CRI file in WPS. [file 1471-2105-7-30-S2.ppt]

## Slide 1
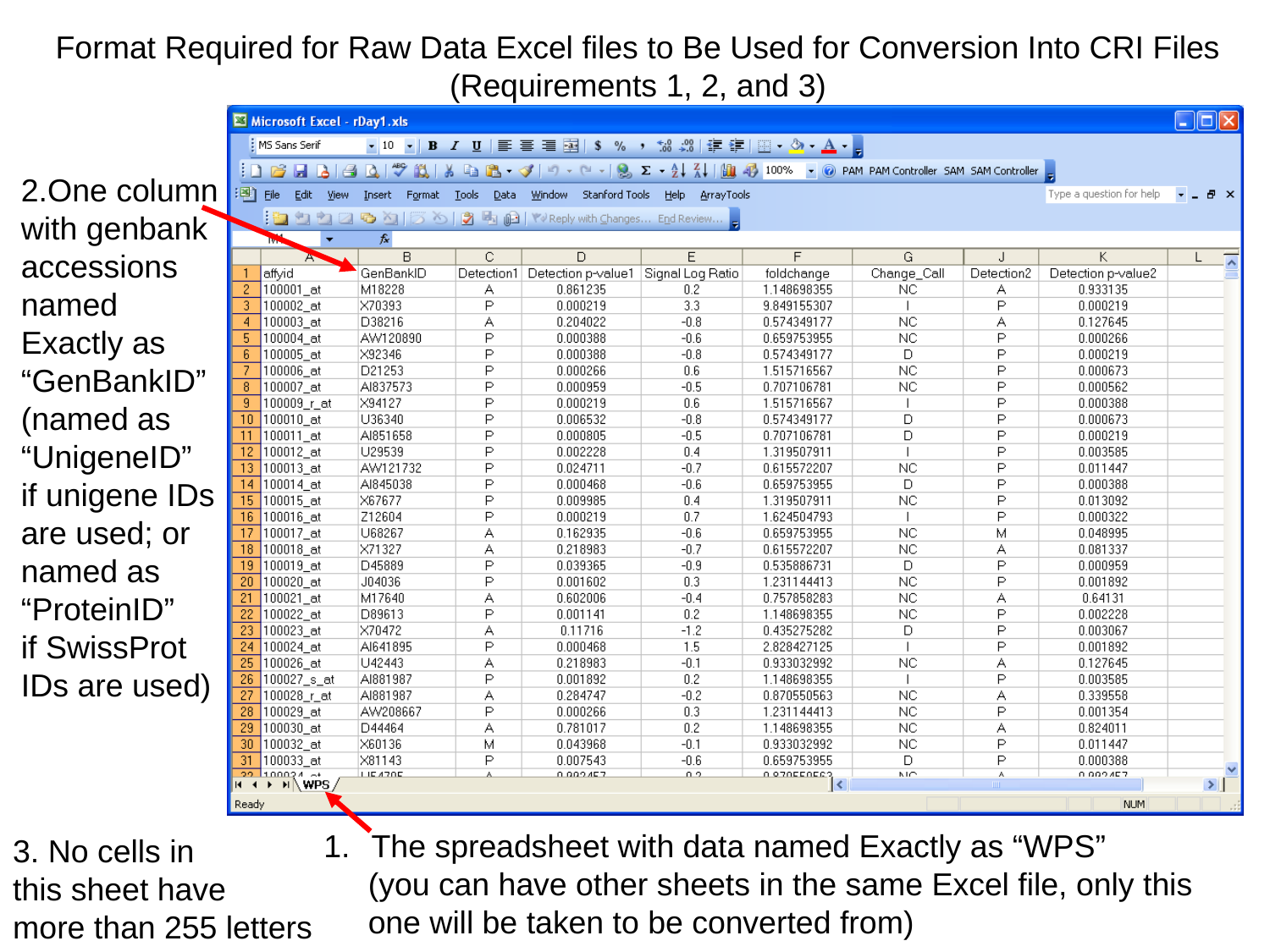

Format Required for Raw Data Excel files to Be Used for Conversion Into CRI Files
(Requirements 1, 2, and 3)
2.One column
with genbank
accessions
named
Exactly as
“GenBankID”
(named as
“UnigeneID”
if unigene IDs
are used; or
named as
“ProteinID”
if SwissProt
IDs are used)
The spreadsheet with data named Exactly as “WPS”
 (you can have other sheets in the same Excel file, only this
 one will be taken to be converted from)
3. No cells in
this sheet have
more than 255 letters
