# Supplementary material for: WholePathwayScope: a comprehensive pathway-based analysis tool for high-throughput data
Source: BMC Bioinformatics. 2006 Jan 19;7:30. doi: 10.1186/1471-2105-7-30 (PMC1388242; doi:10.1186/1471-2105-7-30)
Supplement: Additional File 3 — A Microsoft Word file including a detailed description of the three types of files in WPS. [file 1471-2105-7-30-S3.doc]

***Files to describe individual pathways – PSCP files (local view)***

The overall program layout is described in Figure 1A. Three types of files with different formats have been developed to display the HTP data. The first file type is the PathwayScope File (PSCP). PSCP files contain the identifiers (gene tags) for each gene in a group of related genes or in a pathway. Each gene in a PSCP file has both a GenBank ID and a gene name (if no gene name exists, the GenBank ID is used). The program assigns all GenBankIDs for a single identifier or gene tag, to a BaseGenBankID. Gene tags can be entered manually into a PSCP files or can be entered into the PSCP file from a Microsoft Excel or text file that contains the GenBank IDs and/or gene names.

Graphical presentation of many metabolic pathways and gene groupings are included with the program as PSCP files. These pathways and collections of genes come from three major sources. First, more than 150 pathway HTML files from the KEGG database were downloaded [35]; the GenBank ID numbers for each gene was extracted into individual Excel files using a perl script. The Excel files were then used to create PSCP files containing the genes for each pathway. The pathway layouts were added manually to generate the final PSCP files represented as canonical pathways. In addition, total 280 PSCP files were derived from BioCarta pathway collections of CGAP [36, 37]. A total of 1500 PSCP files were derived from the Gene Ontology (GO) database [38, 39, 40]; the GenBank ID numbers for each gene group were extracted into individual Excel files and used to create PSCP files. To complement these files, ~50 metabolic pathways related to lipid metabolism were manually curated and available for use with the program. Alternatively, compatible internal databases for human and mouse also include these pathways or GO collections as the basis for creating gene-term association network, as described later. The genes of these pathways or GO terms can also be directly retrieved from the internal database to create individual PSCP files. For Biocarta pathways, the pathway graphs can be also visualized in a separate internet browser and data of selected genes in CRI files (see below) can be highlighted in the graphs as well (see additional file 1 for screenshot).

The user can also personalize the arrangement of the gene tags using graphical elements via the program including gene tags, shapes, lines, and text from a Draw Tool Window. Users are encouraged to create additional PSCP files that can be shared through the WPS website [63]. All of the custom PSCP files now available used gene identifiers for murine genes and thus can only be used to analyze HTP data from mice. However, we do provide databases covering these GO terms or pathways for both human and mouse. Users can also create new PSCP files to analyze data from other species.

***Files to describe pathway-pathway relationships – WSCP files (global view)***

The second type of file in the program is the WholeScope File (WSCP file), which is composed of a series of pathway tags. Each pathway tag in the WSCP file is linked to a PSCP file or represents a term (either a pathway or GO term in the internal database). The PCSP file can be accessed if it is linked by a pathway tag or a new PSCP file(s) can be dynamically created if the pathway tag represents a database associated term from the WSCP file by clicking on the pathway tag. Global changes in expression levels of genes in each pathway or term can be indicated by setting criteria in CRI files (see below) to color code the pathway tags. Colors are chosen to indicate changes in expression patterns of genes within the pathway criteria, according to the specification of the user (for example, the tags can be colored red only if the expression level of 3 or more genes or 40% of the total genes in the pathway are increased over 2-fold) . Pathway tags can be added to the PSCP files to link with other pathways or files so that pathways can be networked with each other.

***Criteria files (CRI files) are WPS data files for defining color criteria of data***

Criteria (CRI) files are used to enter the user-defined criteria and HTP data for WPS. Each CRI file is a Microsoft Access file that contains a HTP (e.g. microarray) dataset, the WPS BaseGenBankID for each microarray element, and the user-defined color criteria for the PSCP and WSCP files. HTP datasets are converted to CRI files in the program from Excel files (Microsoft, Inc) containing the HTP data through a Data Conversion Window (Fig. 1B). Three requirements for the dataset to be converted to a CRI file: 1. dataset in one of the work sheets named as “WPS’ in Excel file; 2. Name the column with GenBank Accessions as “GenBankID” (if Unigene IDs, named as “UnigeneID” and if Protein ID, named as “ProteinID” and select appropriate option in mapping options in the “Data Conversion Window” of the program when doing the dataset conversion. 3. Make sure any cells in the dataset worksheet have no more than 255 letters.

Since most microarray or proteomics core facilities or warehouses will provide certain standard gene identifiers (GenBank Accessions, Unigene IDs, Protein IDs (SwissProt) etc.), this form of universal mapping will provide access to the most sources of high throughput data possible. This is because synchronization of these IDs with all of the manufacturers’ platforms is a difficult task, whereas they will all provide some form of a unification key. In addition, Affymetrix and other microarray suppliers already have many ID mapping tables available through their website as well for ID mapping to standard identifiers. In general, our program can be used for any types of microarray data, as long as they are formatted as spreadsheets in Excel files and containing standard or general gene/protein identifiers, (e.g., GenBank Accessions, Unigene IDs, and Protein IDs (SwissProt)). We might add the multiple platform microarray probe IDs coverage in our on-going web-based use of the program in the future if such demand is high. Also, some standards are emerging that will hopefully facilitate this process.

Even if a gene does not have an available ID, as long as the user assigns or defines an ID for it, the gene will still be included in the converted CRI files. The gene can be also used in analysis such as pattern extraction, in which it can be extracted out with its assigned ID as long as its data meets the pattern criteria. In addition, the user has a way to add this gene using its assigned ID into the internal database and create a PSCP or WSCP file including this gene using this ID, so that it will also be colored by the CRI file including it with the assigned ID. This is useful particularly for a new gene that NCBI has not assigned an ID for it, or the users want to handle this gene as their “own” genes.

The HTP data in an Excel file can be an individual dataset, or combined multiple datasets in a single file (i.e. in Stanford format {42]). In the latter case, the user can select an appropriate data column to build color criteria specifically for one dataset in the whole file as individual CRI file, or multiple data columns to build color criteria even for multiple datasets within a single CRI file.

In the case of microarray data, all array elements corresponding to a single gene are mapped to a single identification number, via an internal Microsoft Access database that contains a Unigene-GenBank ID relation table (extracted from the NCBI databases) [31], an annotation table for genes with Unigene IDs, and an annotation table for genes without Unigene IDs. The internal database collates all gene IDs for a given gene and assigns a single BaseGenBankID, which becomes a part of the criteria (CRI) file.

Color criteria for individual gene tags are defined according to the attributes of the HTP data, which usually include parameters describing the relative expression level and data quality. Criteria are specified using logical expressions (>, <, =, AND, OR, NOT, FOLDCHANGE > 3 AND CHANGECALL = 'I') (see Table 1). Color criteria for pathway tags are defined according to the number (or percentage) of genes in the pathway that meet the criteria specified for the individual gene tag (Fig. 1B).

The CRI files containing the microarray or other HTP data and selection criteria are then loaded into the WPS program. Multiple CRI files, corresponding to multiple HTP experiments, can be analyzed simultaneously. WPS first sorts the HTP data into groups according to the criteria specified by the user. WPS then retrieves the first gene in the pathway (PSCP), determines which set of criteria it meets, and then assigns it the corresponding color code. This process is then repeated sequentially for each gene in the pathway until every gene has been assigned a color code. WPS then assigns a color code to each pathway in the file according to the number (or percentage) of genes in the pathway that are up- or down-regulated, as defined by the user. Multiple pathways can be analyzed and displayed at one time (see results). If data from multiple HTP datasets are displayed on the same pathway, both pathway and gene tags are viewed as discrete boxes (where each box represents data from one experiment). WPS also provides the option of using a gradient of colors to reflect the gene expression levels by the intensity of color (not shown).

Since many genes are detected by multiple probesets that co-exist in a dataset in many microarray platforms, and their behaviors can be quite different. In many cases, this reflects splicing variation under different physiological conditions but could also reflect bad probeset design. It becomes critical how these are handled. WPS handles such cases in multiple levels: 1) virtually remove “bad” probesets during CRI criteria setting using quality call as a criteria in addition to quantity data, 2) use hierarchical method to prioritize probesets and use first matching criteria as the call for gene, 3) give user access to all probesets for a particular gene in popup Data Details window, allowing them to be aware of conflicts and apply their own handling rules, and 4) include each gene only once in Fisher’s exact test evaluations. Together these steps give the user considerable flexibility without sacrificing any data in this important and evolving aspect of microarray analysis.
