# Supplementary material for: WholePathwayScope: a comprehensive pathway-based analysis tool for high-throughput data
Source: BMC Bioinformatics. 2006 Jan 19;7:30. doi: 10.1186/1471-2105-7-30 (PMC1388242; doi:10.1186/1471-2105-7-30)
Supplement: Additional File 4 — A Microsoft PowerPoint file including a few slides of screenshots to describe the feature for pattern extraction of genes from a colored PSCP file. Slide1: A colored PSCP file (previously has been loaded with CRI files) subjected to pattern extraction. Slide 2: The pattern extraction window for extraction of genes from the colored PSCP file in slide 1 that match with the defined color pattern in the color template panel. Slide 3: The created PSCP file including the extracted genes in slide 2 to verify the pattern of extracted genes colored with same set of CRI files. [file 1471-2105-7-30-S4.ppt]

## Slide 1
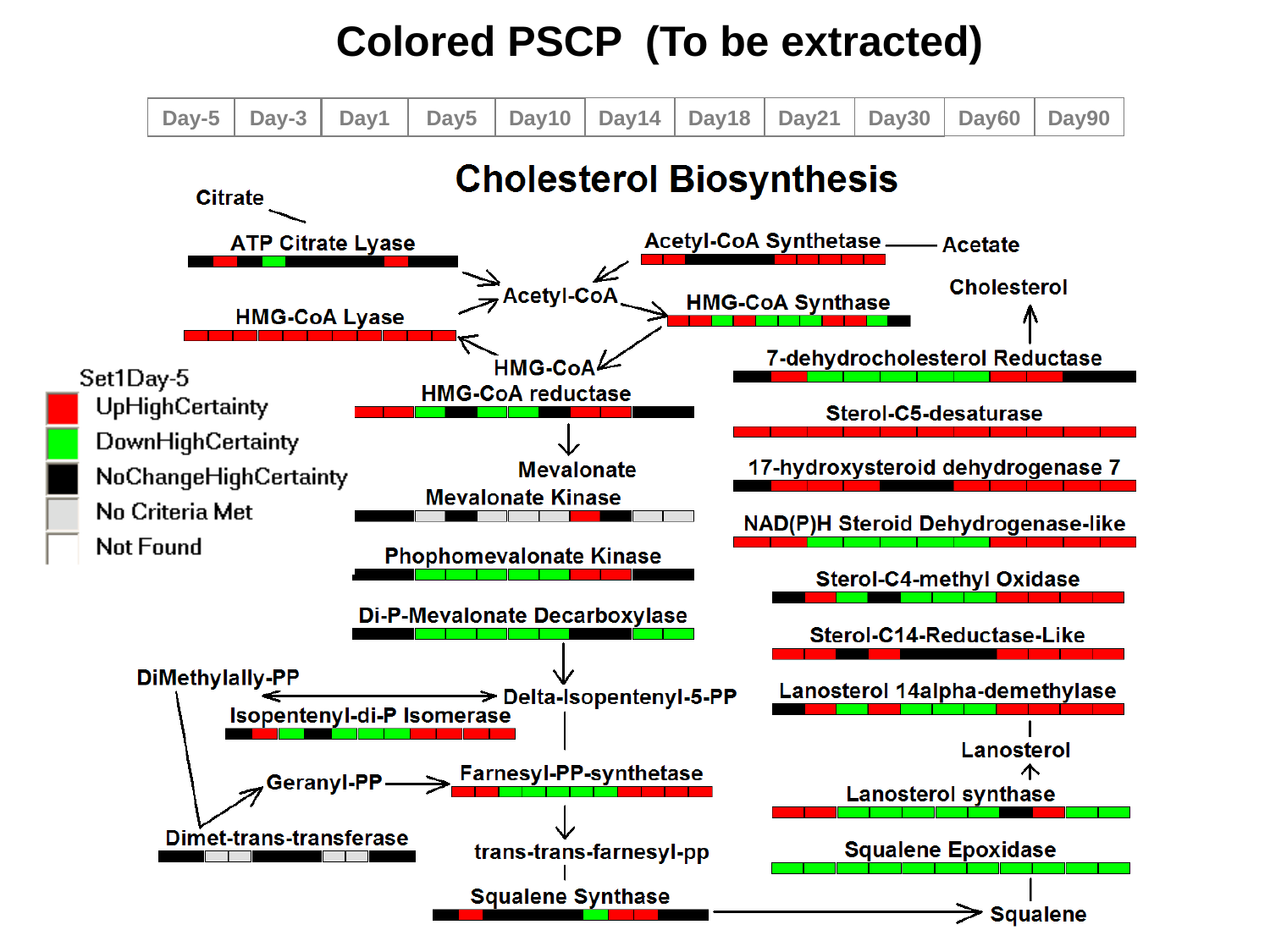

Colored PSCP (To be extracted)
Day60
Day90
Day14
Day18
Day21
Day30
Day-5
Day-3
Day1
Day5
Day10

## Slide 2
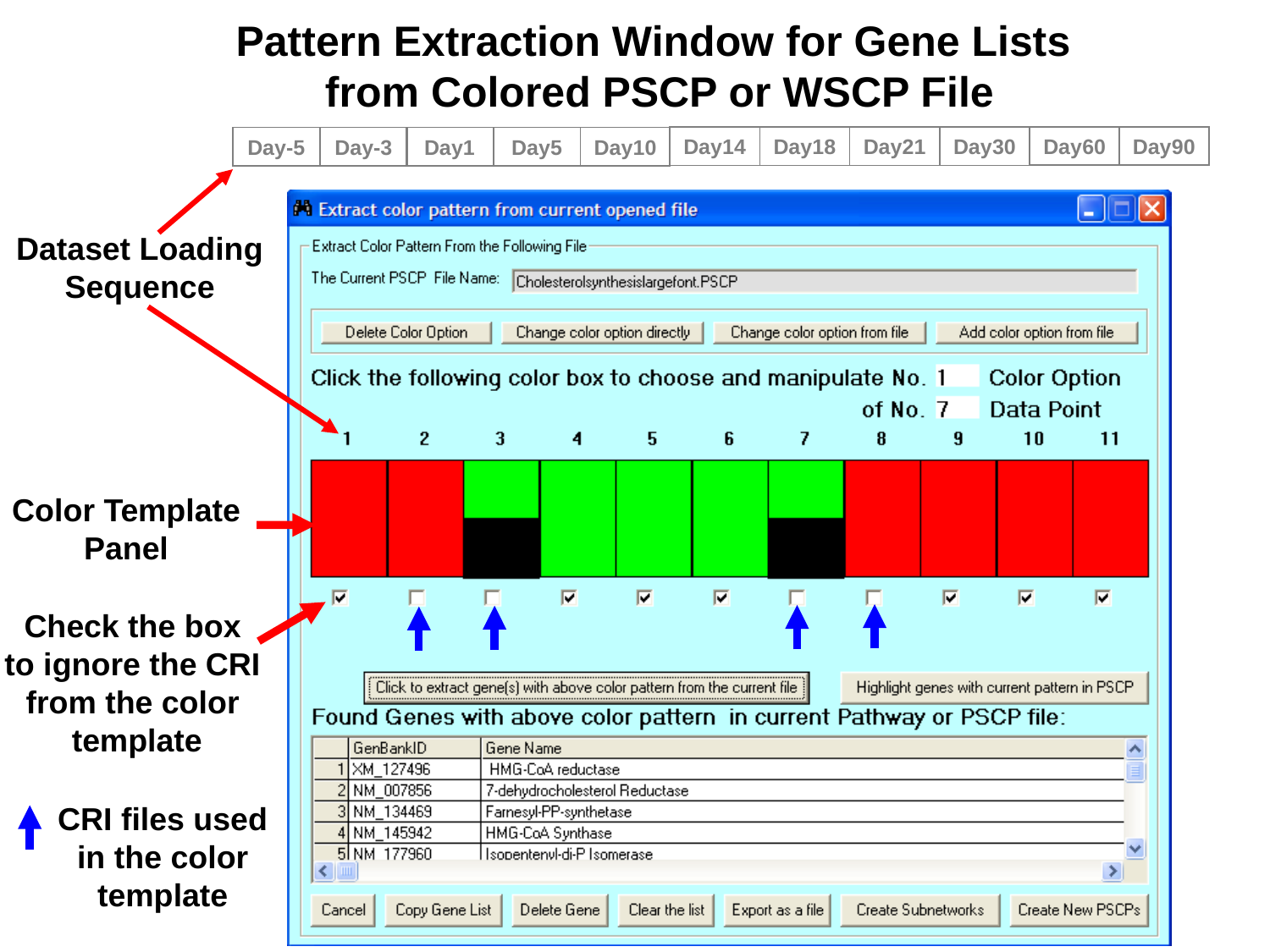

Pattern Extraction Window for Gene Lists
from Colored PSCP or WSCP File
Day60
Day90
Day14
Day18
Day21
Day30
Day-5
Day-3
Day1
Day5
Day10
Dataset Loading
Sequence
Color Template
Panel
Check the box
to ignore the CRI
from the color
template
CRI files used
in the color
template

## Slide 3
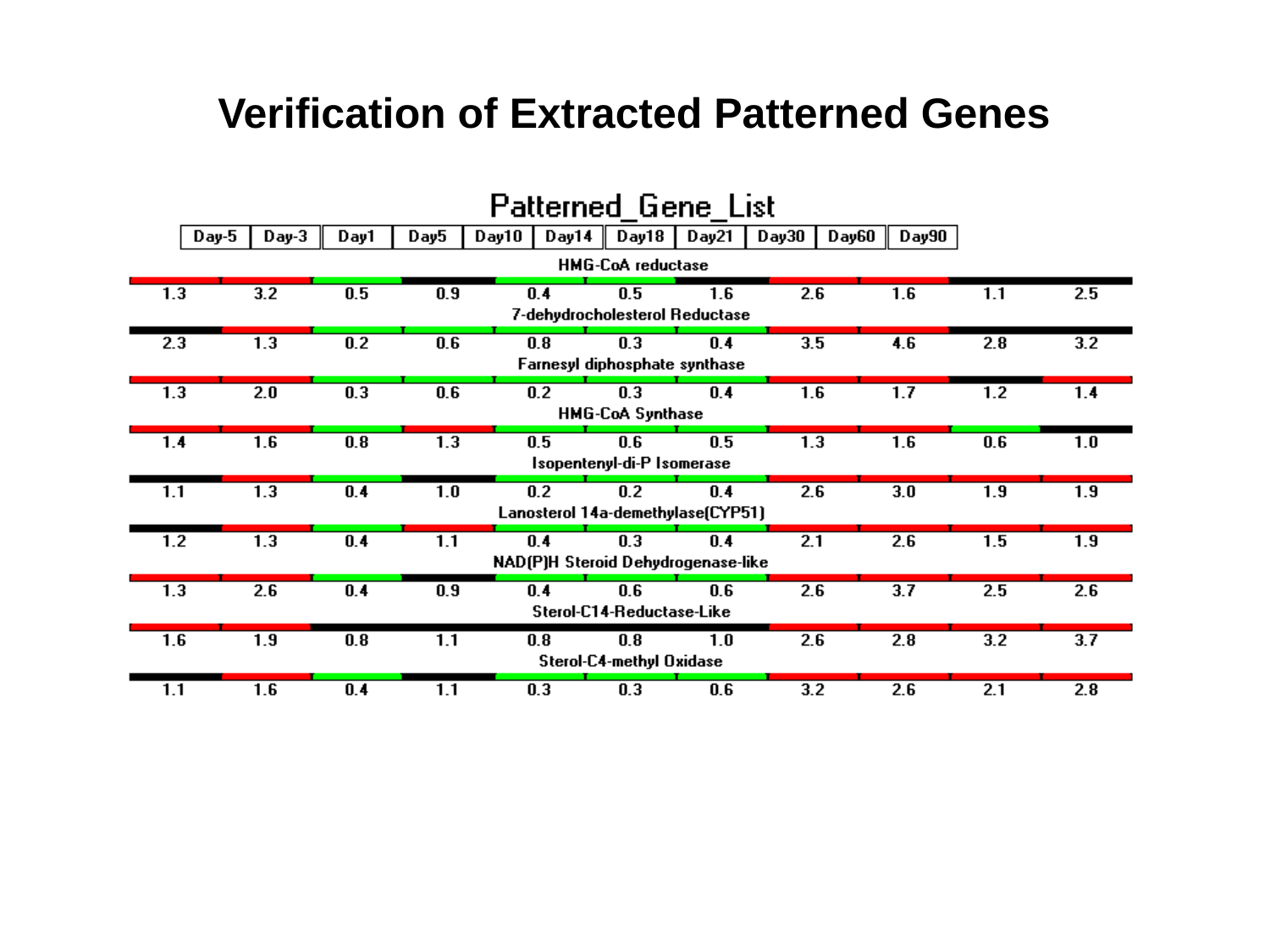

Verification of Extracted Patterned Genes
