# Supplementary material for: WholePathwayScope: a comprehensive pathway-based analysis tool for high-throughput data
Source: BMC Bioinformatics. 2006 Jan 19;7:30. doi: 10.1186/1471-2105-7-30 (PMC1388242; doi:10.1186/1471-2105-7-30)
Supplement: Additional File 5 — A Microsoft Word file including description of the feature for manipulation and filtering of GTANs. [file 1471-2105-7-30-S5.doc]

***Generation and manipulation of Gene-Term Association Network (GTAN) to explore gene-pathway or gene-term relations***

Using an input or filtered gene list, such as lists of genes derived from clustering analysis from other programs or pattern extraction in WPS, the associated pathways or GO terms in the internal database can be identified. These results are listed into the result table in a gene-term pairwise format. Then, the pairwise relationships between genes and their associated pathways or GO terms in the results table can be used to generate a gene-term association network (GTAN) within a PSCP file and is illustrated in a graphical view. The generation of such a network is based on the Scalable Vector Graphics (SVG) technology [43], a standard for describing the layout of two-dimensional graphics in XML. Each gene is represented by a gene tag and its associated pathway/term represented as a pathway tag is linked to the gene with a line. Within a network, when a gene tag is moved, the linked line will follow to maintain the relationship.

The gene-term association network can be manipulated and filtered for the purpose of different analysis needs in many ways including: (1). Filter by gene-term association degree (Fig. 4C); (2). Filter based on Fisher’s exact test results (Fig. 4A, 4B); (3). Filter or Retrieve orphan nodes (genes and/or terms); (4). Retrieve a subnetwork of selected or highlighted nodes in the network (Fig. 5B) (5). Convert to gene-gene or term-term network (Fig. 5A). In addition, genes associated with disease terms, which were derived from Genetic Association Database and MedGene Database and included in the internal database described above, can be highlighted and selected from the network for further analysis and network manipulation (see additional file 6 for screenshot).
