# Supplementary material for: WholePathwayScope: a comprehensive pathway-based analysis tool for high-throughput data
Source: BMC Bioinformatics. 2006 Jan 19;7:30. doi: 10.1186/1471-2105-7-30 (PMC1388242; doi:10.1186/1471-2105-7-30)
Supplement: Additional File 6 — A Microsoft PowerPoint file including a slide for screenshot of the window for searching network for specific genes or terms or for disease-associated genes. The selected disease from database is used to search and highlight the associated genes in current GTAN/PSCP file for further analysis. [file 1471-2105-7-30-S6.ppt]

## Slide 1
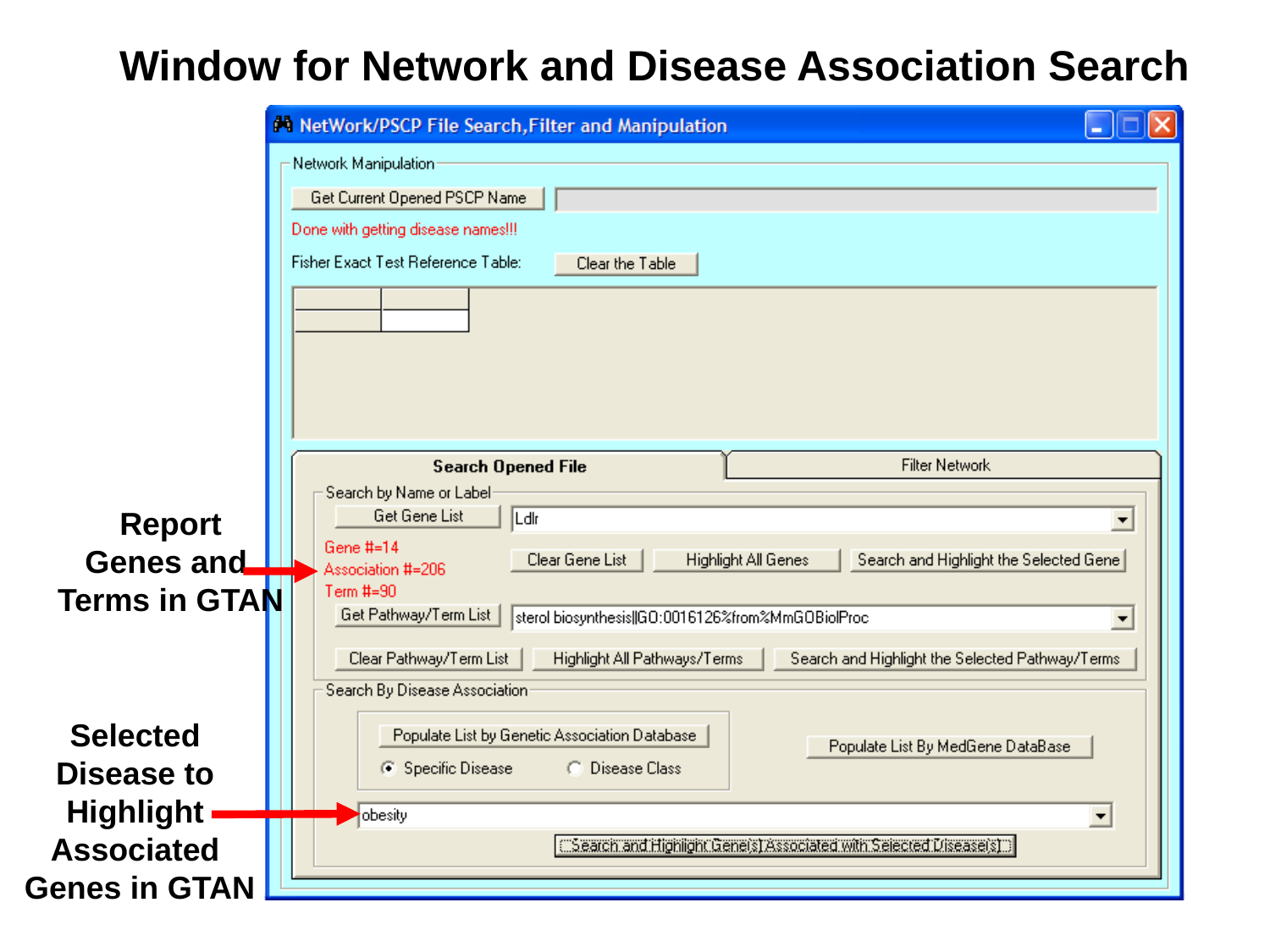

Window for Network and Disease Association Search
Report
Genes and
Terms in GTAN
Selected
Disease to
Highlight
Associated
Genes in GTAN
