# Supplementary material for: WholePathwayScope: a comprehensive pathway-based analysis tool for high-throughput data
Source: BMC Bioinformatics. 2006 Jan 19;7:30. doi: 10.1186/1471-2105-7-30 (PMC1388242; doi:10.1186/1471-2105-7-30)
Supplement: Additional File 7 — A Microsoft PowerPoint file including a slide for illustration of a 2 × 2 contingency table used as basis for Fisher's exact test. [file 1471-2105-7-30-S7.ppt]

## Slide 1
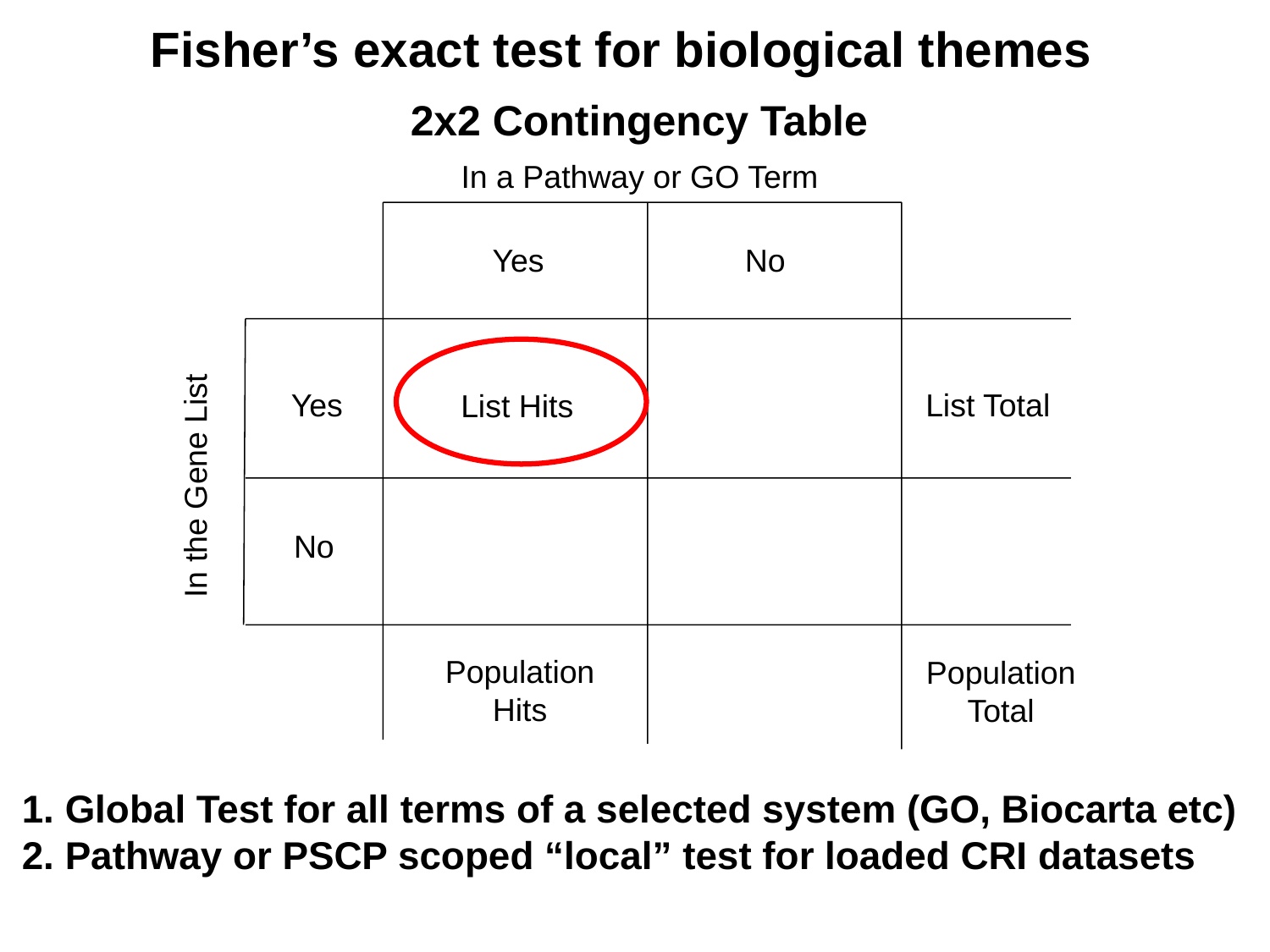

Fisher’s exact test for biological themes
2x2 Contingency Table
In a Pathway or GO Term
Yes
No
Yes
List Total
List Hits
In the Gene List
No
Population
Hits
Population
Total
1. Global Test for all terms of a selected system (GO, Biocarta etc)
2. Pathway or PSCP scoped “local” test for loaded CRI datasets
