# Supplementary material for: WholePathwayScope: a comprehensive pathway-based analysis tool for high-throughput data
Source: BMC Bioinformatics. 2006 Jan 19;7:30. doi: 10.1186/1471-2105-7-30 (PMC1388242; doi:10.1186/1471-2105-7-30)
Supplement: Additional File 9 — A Microsoft PowerPoint file including a few slides of screenshots to describe the feature for pathway or PSCP-scoped "local Fisher's exact test" of user-defined pattern enrichment of choice genes colored with CRI file(s) in a PSCP file being analyzed. Slide1: A colored PSCP file (previously has been loaded with CRI files) subjected to "local Fisher's exact test". Slide 2: The "local Fisher's exact test" window for measuring statistically the enrichment of genes with user-defined criteria, in this example, the enrichment degree of differentiated expressed genes (red and green colors in the color template panel) for each dataset within this pathway. [file 1471-2105-7-30-S9.ppt]

## Slide 1
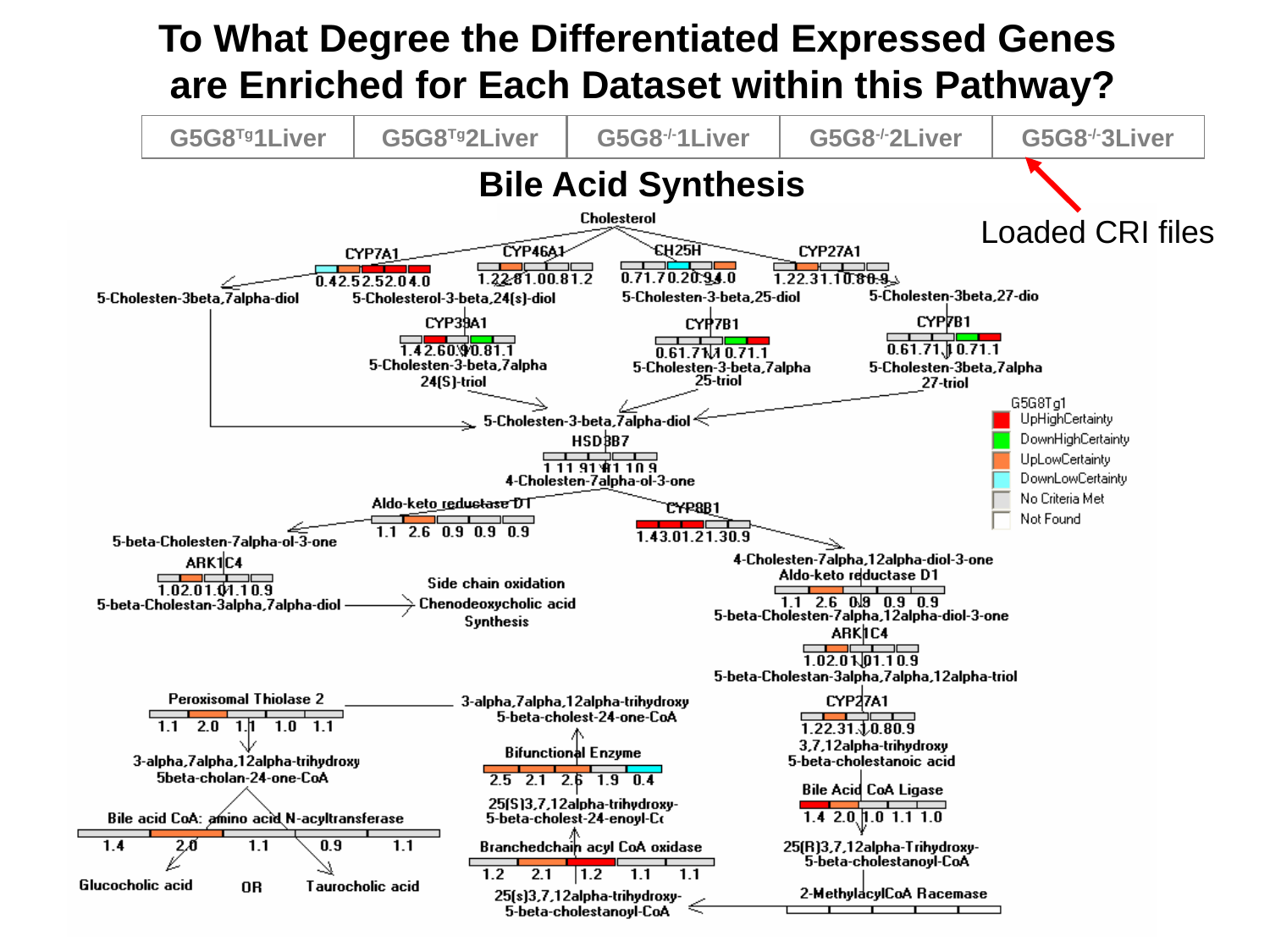

To What Degree the Differentiated Expressed Genes
are Enriched for Each Dataset within this Pathway?
G5G8Tg1Liver
G5G8Tg2Liver
G5G8-/-1Liver
G5G8-/-2Liver
G5G8-/-3Liver
Bile Acid Synthesis
Loaded CRI files

## Slide 2
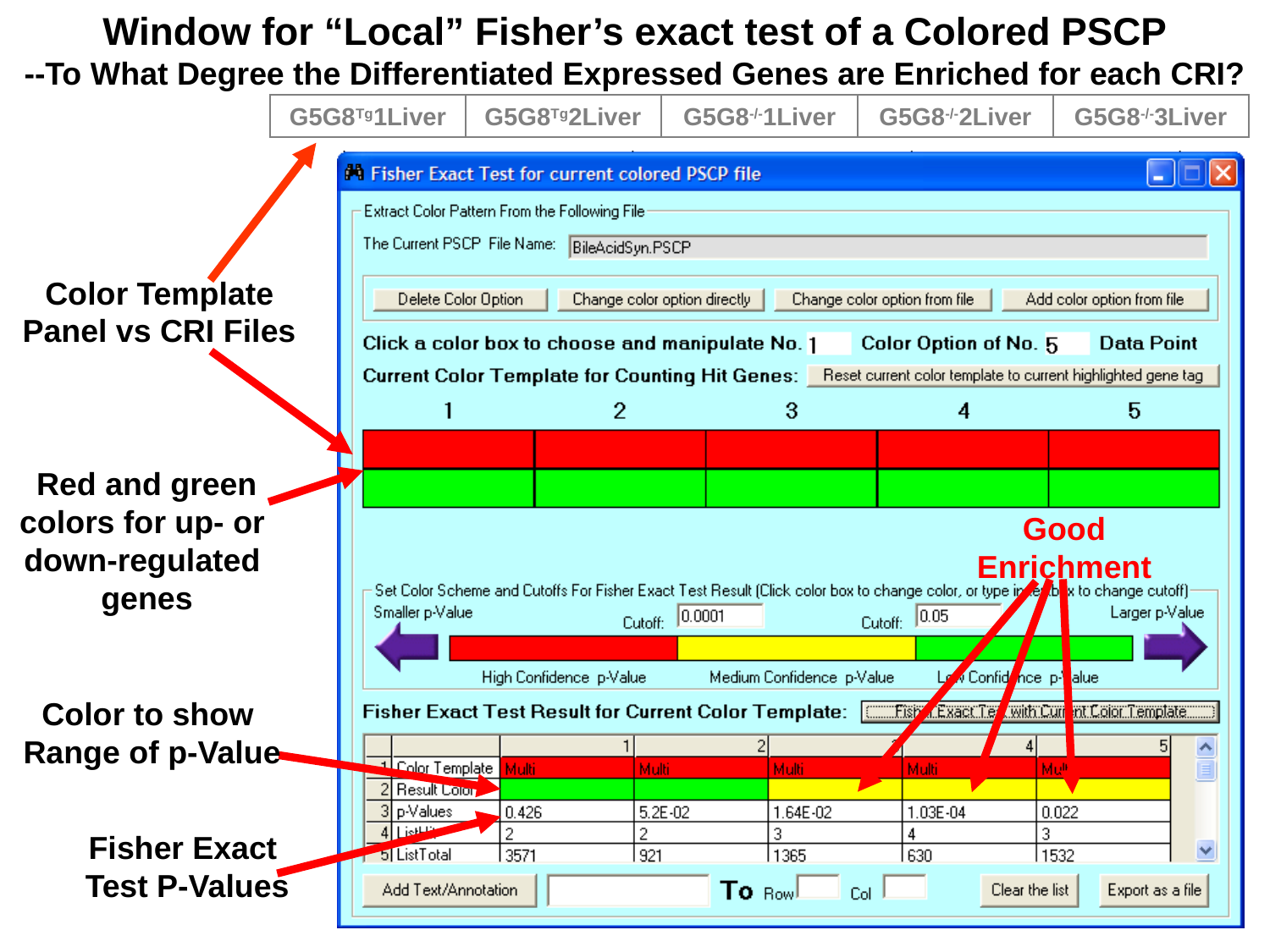

Window for “Local” Fisher’s exact test of a Colored PSCP
--To What Degree the Differentiated Expressed Genes are Enriched for each CRI?
G5G8Tg1Liver
G5G8Tg2Liver
G5G8-/-1Liver
G5G8-/-2Liver
G5G8-/-3Liver
Color Template
Panel vs CRI Files
Red and green
colors for up- or
down-regulated
genes
Good Enrichment
Color to show
Range of p-Value
Fisher Exact
Test P-Values
