# Supplementary material for: WholePathwayScope: a comprehensive pathway-based analysis tool for high-throughput data
Source: BMC Bioinformatics. 2006 Jan 19;7:30. doi: 10.1186/1471-2105-7-30 (PMC1388242; doi:10.1186/1471-2105-7-30)
Supplement: Additional File 10 — A Microsoft PowerPoint file including a slide for screenshot of the window from WPS for sorting a dataset to pathway/term scoped sub-datasets for further processing. [file 1471-2105-7-30-S10.ppt]

## Slide 1
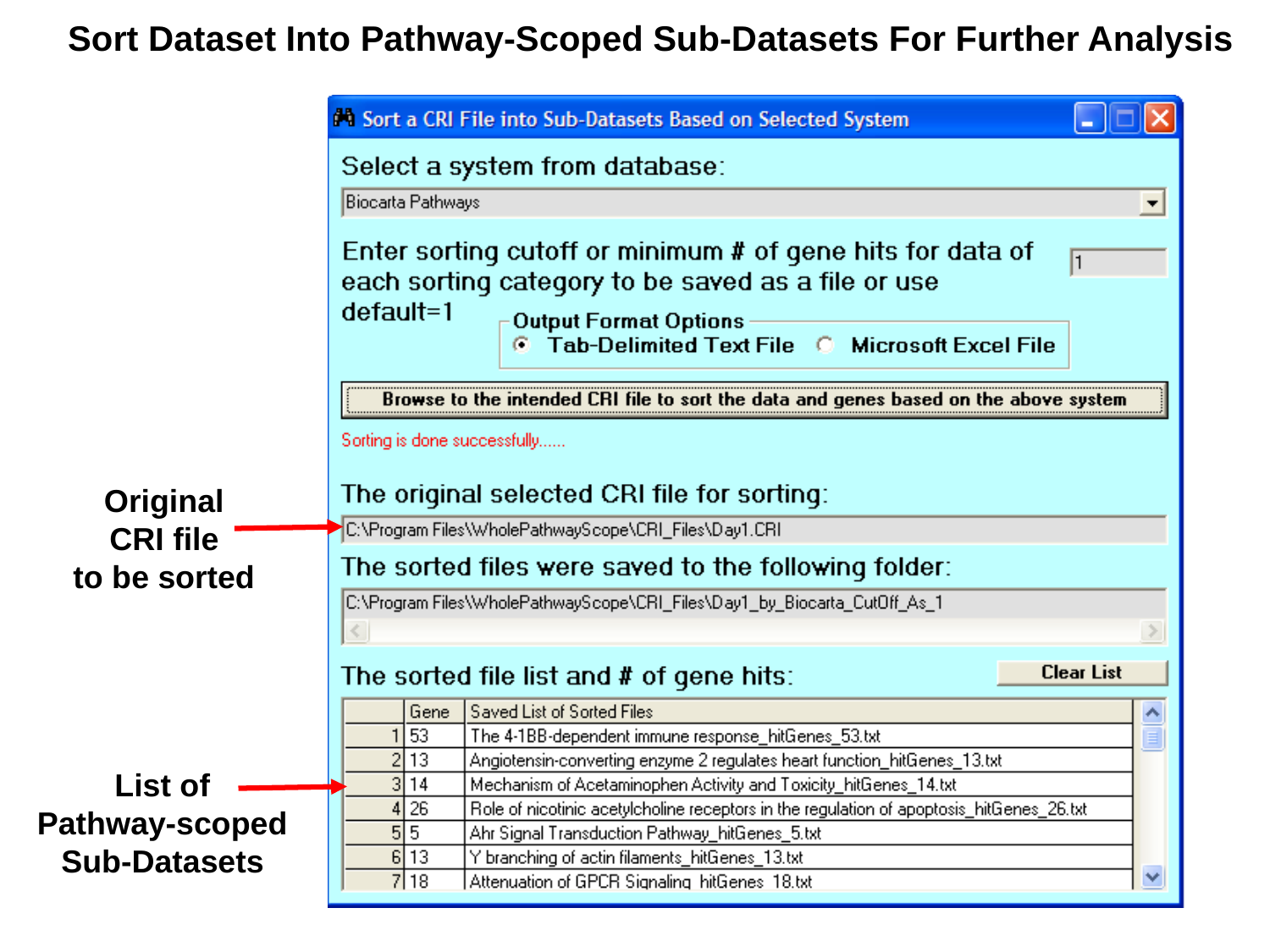

Sort Dataset Into Pathway-Scoped Sub-Datasets For Further Analysis
Original
CRI file
to be sorted
List of
Pathway-scoped
Sub-Datasets
