# Supplementary material for: WholePathwayScope: a comprehensive pathway-based analysis tool for high-throughput data
Source: BMC Bioinformatics. 2006 Jan 19;7:30. doi: 10.1186/1471-2105-7-30 (PMC1388242; doi:10.1186/1471-2105-7-30)
Supplement: Additional File 11 — A Microsoft Word file describing the materials and methods for preparation of microarray data used for describing the program features. [file 1471-2105-7-30-S11.doc]

**Gene-expression data**

Datasets from five independent microarray experiments from two lines of genetically modified mice and age- and gender-matched controls were analyzed using WPS as examples for illustration purposes. Messenger (total) RNA was isolated from the livers of 3-5 female mice (4-6 months of age) expressing 14 copies of a 90 kb fragment containing the entire genes coding for two members of the ATP-binding (ABC) cassette family of transporters, human ABCG5 and ABCG8 (30). Messenger (total) RNA was also isolated from livers and small intestines of 5 female mice (4 to 6 months old) in which the ABCG5 and ABCG8 genes had been inactivated by homologous recombination (43). All mice were maintained on a 12 h light/12 h dark cycle and sacrificed after a 4 h fast. The mRNA from each experiment was pooled, labeled and incubated with duplicate Affymetrix GeneChips Murine Genome MU 74 A, B, and C. The raw data from each experiment was processed and entered into an Excel file using the Affymetrix MAS 5.0 software.

Datasets from eleven independent microarray experiments from livers of wild-type mice collected at different time points during development were also analyzed. Microarray experiments are performed in similar manner as described above. Liver RNAs were pooled from female mice as follows: two mice each for days -9, -5, 5; four mice for day -3, 10, 14, 18, 21 and 30; 6 mice for day 60 and 90; 12 mice for day 1. The results were expressed relative to day -9.

All these datasets are presented in application examples for purpose of demonstration of WPS usage and features. Therefore, MIAME standard is only included at concise degree to describe these datasets even though all the datasets were designed and performed following the MIAME guideline (http://www.mged.org/Workgroups/MIAME/miame_checklist.html).
