# Supplementary figures and images for: WholePathwayScope: a comprehensive pathway-based analysis tool for high-throughput data
Source: BMC Bioinformatics. 2006 Jan 19;7:30. doi: 10.1186/1471-2105-7-30 (PMC1388242; doi:10.1186/1471-2105-7-30)

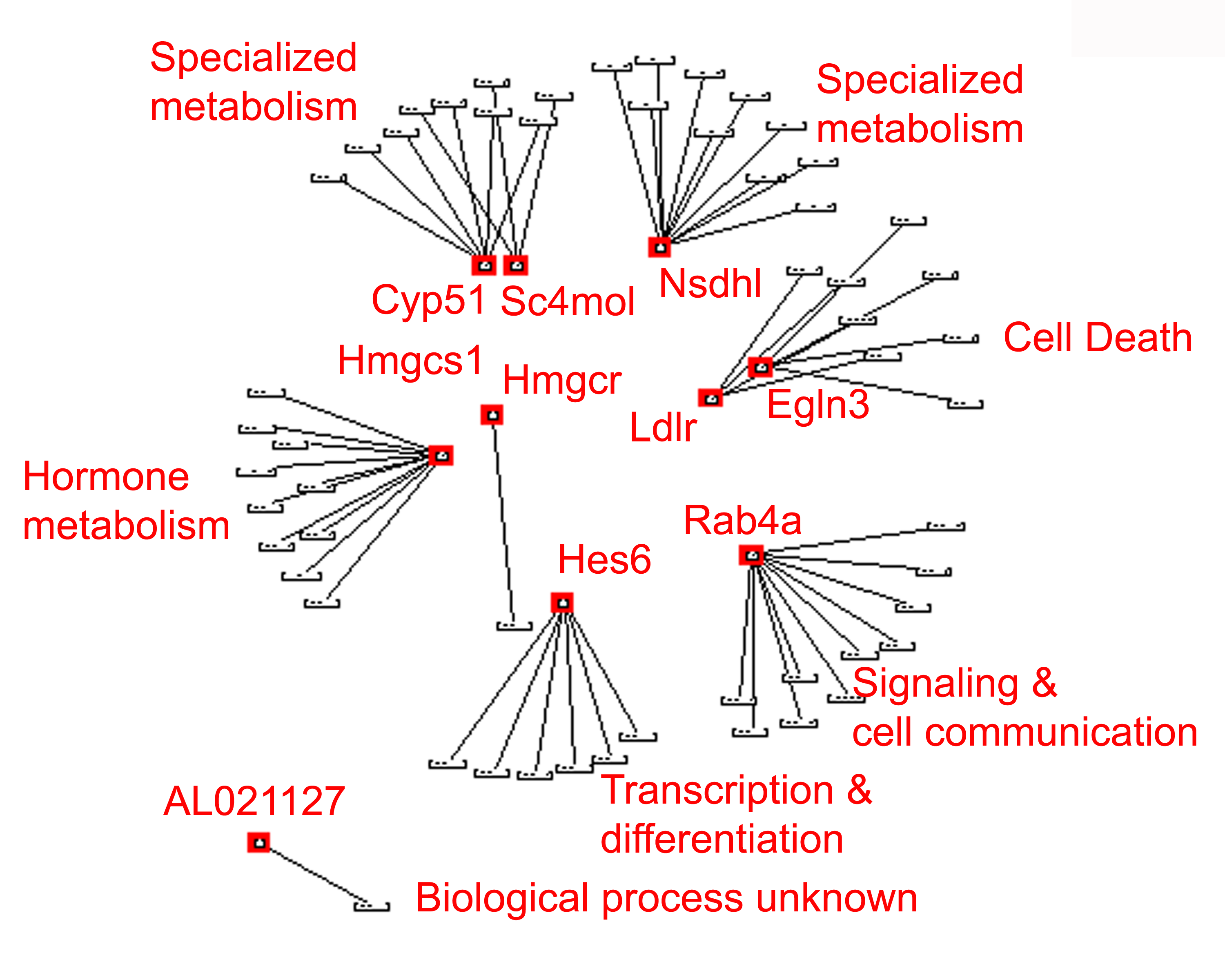

Supplement: Additional File 13 — A graphical tif file to illustrate a filtered GTAN from the GTAN of Fig. 9 for terms with minimal associations of genes, which tend to be unique or specific for their associated genes. Genes are highlighted in red. [file 1471-2105-7-30-S13.tiff]

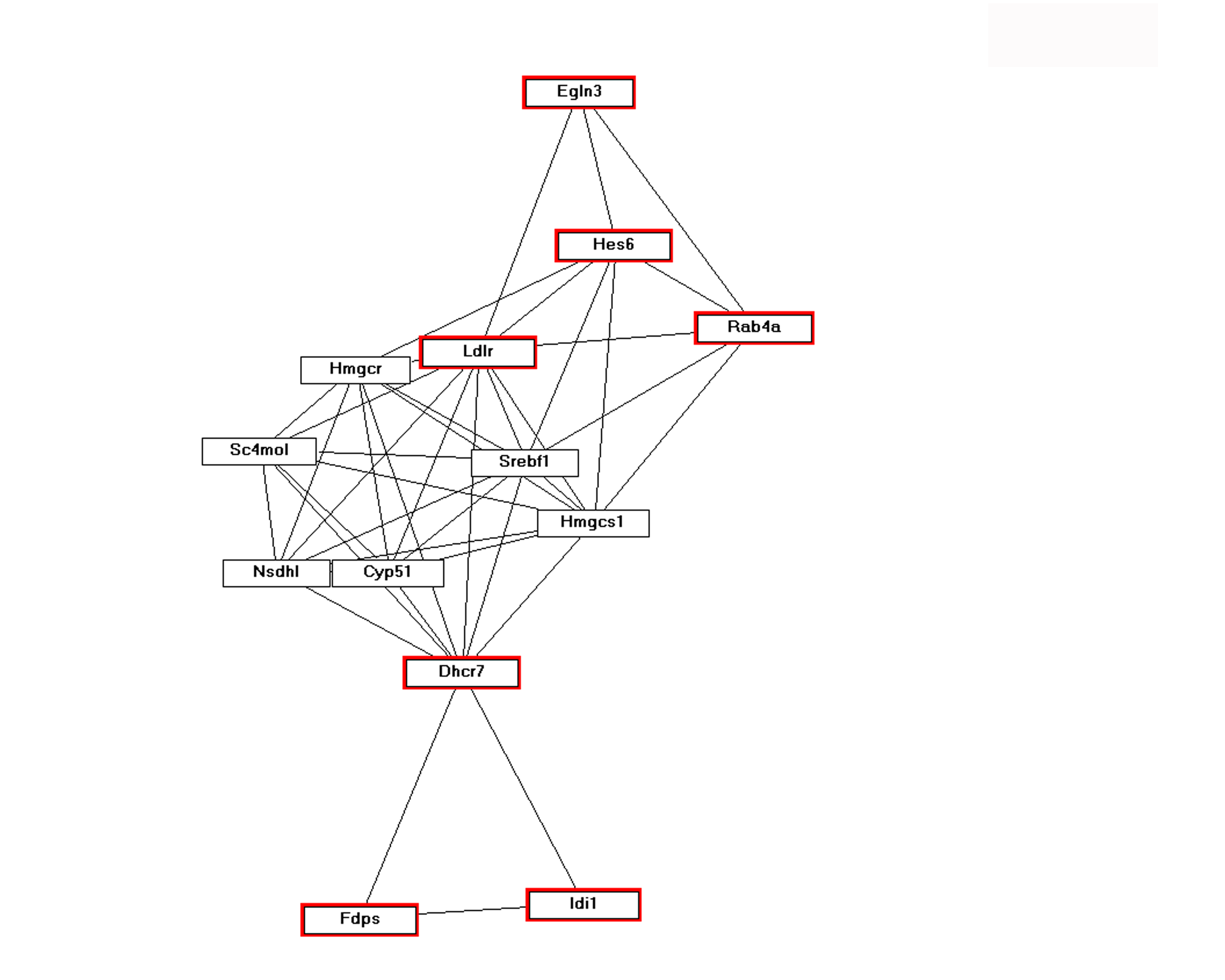

Supplement: Additional File 14 — A graphical tif file to illustrate a GTAN derived from the GTAN of Fig. 12 by merging into a gene-gene network through shared terms. Some genes are highlighted in red used for further manipulation. [file 1471-2105-7-30-S14.tiff]
